# Effects of oral contraceptives on metabolic parameters in adult premenopausal women: a meta-analysis

**DOI:** 10.1530/EC-20-0423

**Published:** 2020-09-10

**Authors:** Lina S Silva-Bermudez, Freddy J K Toloza, Maria C Perez-Matos, Russell J de Souza, Laura Banfield, Andrea Vargas-Villanueva, Carlos O Mendivil

**Affiliations:** 1 School of Medicine, Universidad de los Andes, Bogotá, Colombia; 2 Department of Health Research Methods, Evidence, and Impact, McMaster University, Ontario, Canada; 3 School of Medicine, Universidad de los Andes, and Fundación Santa Fe de Bogotá, Section of Endocrinology, Bogotá, Colombia

**Keywords:** oral contraceptive, lipids, lipoproteins, insulin resistance metabolism

## Abstract

**Objective:**

To estimate the effect of oral contraceptives (OC) containing different progestins on parameters of lipid and carbohydrate metabolism through a systematic review and meta-analysis.

**Patients and methods:**

Premenopausal women aged 18 or older, who received oral contraceptives containing chlormadinone, cyproterone, drospirenone, levonorgestrel, desogestrel, dienogest, gestodene or norgestimate, for at least 3 months. Outcome variables were changes in plasma lipids, BMI, insulin resistance and plasma glucose. We searched MEDLINE and EMBASE for randomized trials and estimated the pooled within-group change in each outcome variable using a random-effects model. We performed subgroup analyses by study duration (<12 months vs ≥12 months) and polycystic ovary syndrome (PCOS) status.

**Results:**

Eighty-two clinical trials fulfilled the inclusion criteria. All progestins (except dienogest) increased plasma TG, ranging from 12.1 mg/dL for levonorgestrel (*P* < 0.001) to 35.1 mg/dL for chlormadinone (*P* < 0.001). Most progestins also increased HDLc, with the largest effect observed for chlormadinone (+9.6 mg/dL, *P* < 0.001) and drospirenone (+7.4 mg/dL, *P* < 0.001). Meanwhile, levonorgestrel decreased HDLc by 4.4 mg/dL (*P* < 0.001). Levonorgestrel (+6.8 mg/dL, *P* < 0.001) and norgestimate (+11.5 mg/dL, *P* = 0.003) increased LDLc, while dienogest decreased it (–7.7 mg/dL, *P* = 0.04). Cyproterone slightly reduced plasma glucose. None of the progestins affected BMI or HOMA-IR. Similar results were observed in subgroups defined by PCOS or study duration.

**Conclusion:**

Most progestins increase both TG and HDLc, their effect on LDLc varies widely. OC have minor or no effects on BMI, HOMA-IR and glycemia. The antiandrogen progestins dienogest and cyproterone displayed the most favorable metabolic profile, while levonorgestrel displayed the least favorable.

## Introduction

Four of every five reproductive-age women in the world have used oral contraceptives (OC) (1). Most OC combine one estrogen with one progestin so there are multiple possible combinations and dosing schemes. Although OC are highly effective for preventing pregnancy, their impact on lipid, lipoprotein, and carbohydrate metabolism is not fully acknowledged. First- and second-generation progestins (desogestrel, gestodene, norgestimate, levonorgestrel, and others) are chemically related to testosterone and may have been undesirable androgenic effects (2). Newer progestins derived from progesterone or spironolactone (cyproterone, chlormadinone, nomegestrol, drospirenone) are expected to result in a more favorable metabolic profile (2).

Estrogens and progestins bind rapidly to nuclear receptors that ultimately regulate the transcription of target genes (2). Estrogens promote insulin secretion, peripheral glucose utilization, synthesis of triglycerides, secretion of HDL, and favor LDL cholesterol uptake and catabolism (3). On the other hand, progesterone induces insulin resistance and hyperglycemia, resembling the physiological state of pregnancy (4). Of note, progestins may bind not only the progesterone receptor, but also the glucocorticoid, mineralocorticoid and androgen receptors with different affinities (3). Androgen receptor binding may induce weight gain and higher plasma LDL cholesterol (2). Combined OC containing newer progestins like drospirenone and dienogest are considered anti-androgenic (3).

Given that available studies have used combined OC with distinct combinations and doses of estrogen and progestin, using a wide variety of comparators, the overall impact of each OC on metabolic variables is not easy to assess. For these reasons, we conducted a pre–post effect size meta-analysis of randomized clinical trials in order to estimate a consolidated effect of OC containing different progestins on plasma lipid profile, body weight, glycemic levels, and insulin resistance, in adult premenopausal women.

## Methods

This meta-analysis was designed and executed according to the guidelines for the preferred reporting items (PRISMA) (5). The questions to be answered by this meta-analysis were: Among premenopausal women: (i) What are the within-person effects of OCs containing different progestins on plasma lipids? (ii) What are the within-person effects of OCs containing different progestins on other metabolically relevant variables (BMI, FPG, HOMA-IR)? (iii) Do the effects of OCs with different progestins differ in women with PCOS vs without PCOS and (iv) Do the effects of OCs on metabolic variables vary by duration of use?

The studies considered for inclusion were those in premenopausal women aged 18 or older, who received oral contraceptives containing chlormadinone, cyproterone, drospirenone, levonorgestrel, desogestrel, dienogest, gestodene or norgestimate, for at least 3 months. The comparator was the baseline value for each outcome variable, namely LDLc, triglycerides (TG), HDLc, HOMA-IR, BMI or fasting plasma glucose (FPG).

### Literature search

The search strategy was devised using a combination of keywords and database-specific controlled vocabulary for the concepts of oral contraceptives, lipids and carbohydrates. Additional terminology was added for randomized clinical trials and to eliminate animal studies. Each database, OVID Medline, OVID Embase, LiLACS, and SciELO, was searched from inception, current to July 2020. Please see Supplementary Table 1 (see section on supplementary material given at the end of this article) for a copy of the OVID Medline search. In addition, the reference lists of prior reviews from the Cochrane Database of Systematic Reviews were also reviewed for relevant citations that may not have been picked up through the search strategy.

The protocol for this review was registered on PROSPERO (CRD42017078740) and can be accessed at: https://www.crd.york.ac.uk/prospero/display_record.php?RecordID=78740.

### Inclusion and exclusion criteria

We included all randomized clinical trials of OC reporting mean and standard deviation of plasma LDL cholesterol, HDL cholesterol or triglycerides before and after treatment. The duration of treatment had to be at least 3 months. Only studies in premenopausal women (with or without PCOS) were included. Studies of hormonal replacement therapy, or studies in which OC were used as a treatment for endometriosis were excluded. Trials with incomplete data reporting were also excluded. The inclusion of only randomized clinical trials allowed us to analyze studies with greater methodologic rigor, provision of study intervention and tracking of adherence, all of which improve the ascertainment of exposure status and increase the internal validity of our results.

### Data collection and risk of bias assessment

Two individual reviewers examined each article for inclusion according to patient characteristics, design, intervention and outcomes. Any disagreement was resolved through discussion and consensus. One record of each study was included in case of duplicates. We retrieved data from each trial in a de-identified manner, using a standardized form that included estrogen and progestin received, number of participants in each group, relevant demographics and length of follow-up. We extracted for each study group the mean and standard deviation of the baseline and final values for our study outcomes: LDL cholesterol (LDLc), triglycerides, HDLc, homeostasis model assessment-insulin resistance (HOMA-IR), BMI and fasting plasma glucose (FPG). Risk of bias was individually evaluated according to the Cochrane Collaboration’s Risk of Bias Assessment Tool. Each study was considered to have risk of bias (yes or no) in each of six determined categories: allocation concealment, blinding of participants and personnel, blinding of outcome assessment, incomplete outcome data, selective reporting, and other bias. We classified studies as follows: If 4–6 domains were ‘yes’: high risk of bias, 2–3 domains: medium risk of bias, 0–1 domains: low risk of bias.

We assessed publication bias by visual assessment of the funnel plot asymmetry, and by performing Begg’s and Egger’s tests when there were at least 5 groups for that particular outcome. In cases in which publication bias was likely, we used the trim and fill method (employing the meta trimfill command in STATA) to correct it.

### Statistical analysis

A meta-analysis was used to compare changes in metabolic outcomes associated with the use of OC containing different progestins. The effect measure for the meta-analysis was the unstandardized pooled mean difference (pMD) (where mean difference = final mean of outcome − baseline mean of outcome) for each variable of interest within each study group. For our analyses, we grouped OC containing the same progestin, regardless of the dose. We quantified the degree of inter-study heterogeneity with the *I*^2^ statistic and its associated CI. The CI for *I*^2^ in most of our study outcomes did not include zero, so we decided to analyze all endpoints using a random-effects model.

Given the heterogeneity between studies in the duration of treatment and study groups, we performed two separate subgroup analyses. First, we performed a comparison by the duration of treatment (less vs more than 12 months), and second, a comparison by the presence of PCOS diagnosis (with PCOS vs without PCOS). In addition, a meta-regression of each outcome vs age and BMI was done for each progestin, adjusting for estrogen dose.

Meta-analyses were executed in RevMan, version 5.0, meta-regression analyses in SPSS, version 23, and trim-and-fill analyses in STATA, version 16.

## Results

We included in this review 143 study groups from 82 studies, published between 1979 and July 2020 (Table 1). Of the 82 studies, 53 (64.6%) were conducted in Europe, the average group sample size was 22 (Q1−Q3: 15−30) and the duration ranged from 3 to 24 months. A total of 2354 women were included, their average age was 25.1 and their mean BMI 24.7 kg/m^2^.

**Table 1:** Characteristics of study groups included in the meta-analysis.

Progestin in OC	First author	Ref.	Country	*n*^a^	Age	BMI	Follow-up (months)	Risk of bias
Chlormadinone	Vieira	(6)	Brazil	20	25.0	23.3	12	Medium
Chlormadinone	Cagnacci	(7)	Italy	12	28.1	23.1	6	Low
Cyproterone	Vrbikova	(8)	Czech Republic	12	25.4	24.4	3	Medium
Cyproterone	Vexiau	(9)	France	24	25.0	20.6	12	Medium
Cyproterone	Vermeulen	(10)	Belgium	13	–	–	6	Low
Cyproterone	Vermeulen	(10)	Belgium	17	–	–	6	Low
Cyproterone	Venturoli	(11)	Italy	20	22.9	22.6	12	Medium
Cyproterone	Teede	(12)	Australia	26	33.5	35.8	6	Medium
Cyproterone	Sabuncu	(13)	Turkey	14	28.8	37.8	6	Medium
Cyproterone	Rautio	(14)	Finland	10	–	–	6	Medium
Cyproterone	Porcile	(15)	Chile	10	25.0	23.2	24	Low
Cyproterone	Moran	(16)	Australia	30	36.0	36.0	6	Medium
Cyproterone	Miccoli	(17)	Italy	20	25.0	21.2	6	Low
Cyproterone	Mhao	(18)	Iraq	10	–	30.5	3	High
Cyproterone	Luque-Ramirez	(19)	Spain	15	23.4	29.2	6	Medium
Cyproterone	Lemay	(20)	Canada	7	20.0	33.9	6	High
Cyproterone	Kahraman	(21)	Turkey	26	21.0	22.8	12	Medium
Cyproterone	Hutchison	(22)	Australia	19	34.1	35.3	6	Medium
Cyproterone	Hagag	(23)	Israel	70	21.0	23.5	12	High
Cyproterone	Fugère	(24)	Canada	40	22.7	22.1	12	Low
Cyproterone	Fugère	(24)	Canada	33	23.2	22.2	12	Low
Cyproterone	Feng	(25)	China	41	28.6	27.8	3	Medium
Cyproterone	Elter	(26)	Turkey	20	23.5	21.8	4	Low
Cyproterone	Dardzińska	(27)	Poland	24	24.9	24.9	4	Medium
Cyproterone	Cetinkalp	(28)	Turkey	33	–	24.7	4	High
Cyproterone	Bilgir	(29)	Turkey	20	24.3	28.2	3	Medium
Cyproterone	Leelaphiwat	(30)	Thailand	16	26.9	23.0	3	Low
Cyproterone	Behboudi-Gandevani	(86)	Iran	32	24.2	25.4	3	High
Cyproterone	Song	(87)	China	60	27.7	28.6	3	High
Desogestrel	März	(31)	Germany	22	–	–	3	Low
Desogestrel	Bertolini	(32)	Italy	20	–	–	6	Low
Desogestrel	Miccoli	(17)	Italy	19	26.0	21.1	6	Low
Desogestrel	Gevers	(33)	Netherlands	28	–	–	12	Low
Desogestrel	März	(34)	Germany	11	–	–	12	Low
Desogestrel	Robinson	(35)	England	17	38.2	20.9	6	Low
Desogestrel	Steinmetz	(36)	Germany	23	21.5	21.1	3	Low
Desogestrel	Petersen	(37)	Denmark	15	24.0	–	3	Low
Desogestrel	Porcile	(38)	Chile	9	–	–	24	Medium
Desogestrel	Porcile	(38)	Chile	6	–	–	24	Medium
Desogestrel	Porcile	(15)	Chile	10	22.5	22.5	24	Low
Desogestrel	Porcile	(15)	Chile	6	22.4	23.5	24	Low
Desogestrel	Kauppinen-Mäkelin	(39)	Finland	15	–	–	3	Medium
Desogestrel	Song	(40)	China	11	32.2	20.8	3	Medium
Desogestrel	Song	(40)	China	11	32.2	20.8	3	Medium
Desogestrel	Cachrimanidou	(41)	Sweden	13	24.0	–	12	High
Desogestrel	Cachrimanidou	(41)	Sweden	7	24.0	–	12	High
Desogestrel	Kuhl	(42)	Germany	16	–	–	3	Low
Desogestrel	Kuhl	(42)	Germany	16	–	–	3	Low
Desogestrel	Singh	(43)	USA	23	24.9	–	6	Low
Desogestrel	van den Ende	(44)	Netherlands	20	27.5	21.3	3	Low
Desogestrel	van der Mooren	(45)	Netherlands	62	26.3	22.5	6	Low
Desogestrel	Gaspard	(46)	Belgium	25	21.2	21.9	13	Medium
Desogestrel	Klipping	(47)	Netherlands	30	23.7	21.7	6	Medium
Desogestrel	Banaszewska	(48)	Poland	48	24.0	22.3	3	Low
Desogestrel	Cagnacci	(49)	Italy	20	–	24.1	6	Medium
Desogestrel	Cagnacci	(7)	Italy	12	27.8	23	6	Low
Desogestrel	Kriplani	(50)	India	29	22.5	26.1	6	Medium
Desogestrel	Shahnazi	(51)	Iran	68	30.0	28.7	3	Low
Dienogest	Wiegratz	(52)	Germany	25	26.1	21.9	6	Low
Dienogest	Wiegratz	(52)	Germany	25	26.1	21.9	6	Low
Dienogest	Junge	(53)	Germany	30	28.1	23.2	6	Medium
Drospirenone	Gaspard	(46)	Belgium	25	21.5	20.2	13	Medium
Drospirenone	Klipping	(47)	Netherlands	29	23.8	21.8	6	Medium
Drospirenone	Özdemir	(54)	Turkey	32	22.7	24.3	6	Medium
Drospirenone	Yildizhan	(55)	Turkey	72	–	22.9	12	High
Drospirenone	Battaglia	(56)	Italy	19	23.4	25.1	6	Low
Drospirenone	Fruzzetti	(57)	Italy	16	24.3	24.7	6	Medium
Drospirenone	Kriplani	(50)	India	29	22.5	27.6	6	Medium
Drospirenone	Machado	(58)	Brazil	39	27.9	22.5	6	Medium
Drospirenone	Machado	(58)	Brazil	38	27.7	22.3	6	Medium
Drospirenone	Mohamed	(59)	Egypt	245	30.9	25.9	12	Medium
Drospirenone	Klipping	(60)	Netherlands	26	24.8	22.5	12	Medium
Drospirenone	Klipping	(60)	Netherlands	21	24.4	25.5	12	Medium
Drospirenone	Klipping	(60)	Netherlands	28	24.8	22.5	12	Medium
Drospirenone	Romualdi	(61)	Italy	15	22.9	22.06	12	Low
Drospirenone	Romualdi	(61)	Italy	15	21.9	22.7	12	Low
Drospirenone	Kahraman	(21)	Turkey	26	21.5	22.0	12	Medium
Drospirenone	Orio	(62)	Italy	50	26.4	27.0	6	Low
Gestodene	Bertolini	(32)	Italy	20	–	–	6	Low
Gestodene	Kjaer	(63)	Denmark	16	24.7	–	6	Low
Gestodene	Miccoli	(17)	Italy	18	24.0	20.9	6	Low
Gestodene	Gevers	(33)	Netherlands	32	–	–	12	Low
Gestodene	März	(31)	Germany	11	–	–	12	Low
Gestodene	Robinson	(35)	England	20	38.1	22.2	6	Low
Gestodene	Steinmetz	(36)	Germany	21	20.1	20.3	3	Low
Gestodene	Petersen	(64)	Denmark	20	24.5	20.9	6	Low
Gestodene	Petersen	(37)	Denmark	19	23.0	–	3	Low
Gestodene	van der Mooren	(45)	Netherlands	62	26.1	22.1	6	Low
Gestodene	Endrikat	(65)	Germany	35	24.7	22.7	6	Low
Gestodene	Endrikat	(65)	Germany	34	25.2	22.6	6	Low
Gestodene	Merki-Feld	(66)	Switzerland	8	22.0	–	3	Medium
Gestodene	Merki-Feld	(66)	Switzerland	8	20.3	–	3	Medium
Gestodene	Merki-Feld	(67)	Switzerland	6	22.8	21.6	3	High
Gestodene	Merki-Feld	(67)	Switzerland	6	20.8	24.2	3	High
Gestodene	Yildizhan	(55)	Turkey	71	–	22.5	12	High
Levonogestrel	Larsson-Cohn	(68)	Sweden	24	27.3	–	6	Low
Levonogestrel	Larsson-Cohn	(68)	Sweden	20	23.3	–	6	Low
Levonogestrel	Larsson-Cohn	(68)	Sweden	23	25.0	–	6	Low
Levonogestrel	Larsson-Cohn	(68)	Sweden	20	23.3	–	6	Low
Levonogestrel	Larsson-Cohn	(68)	Sweden	23	25.0	–	6	Low
Levonogestrel	Larsson-Cohn	(69)	Sweden	25	–	–	6	High
Levonogestrel	Larsson-Cohn	(69)	Sweden	25	–	–	6	High
Levonogestrel	Larsson-Cohn	(69)	Sweden	24	–	–	6	High
Levonogestrel	Larsson-Cohn	(69)	Sweden	24	–	–	6	High
Levonogestrel	März	(34)	Germany	22	–	–	3	Low
Levonogestrel	Bertolini	(32)	Italy	20	–	–	6	Low
Levonogestrel	Boonsiri	(70)	Thailand	59	23.8	–	12	Medium
Levonogestrel	Boonsiri	(70)	Thailand	62	23.7	–	12	Medium
Levonogestrel	Boonsiri	(70)	Thailand	66	24.5	–	12	Medium
Levonogestrel	Kjaer	(63)	Denmark	17	24.7	–	6	Low
Levonogestrel	Notelovitz	(71)	USA	29	25.4	23.6	12	Low
Levonogestrel	Patsch	(72)	USA	45	–	–	6	Medium
Levonogestrel	Loke	(73)	Singapore	21	25.7	20.8	12	Medium
Levonogestrel	Loke	(73)	Singapore	24	25.3	21.0	12	Medium
Levonogestrel	Steinmetz	(36)	Germany	15	22.5	22.1	3	Low
Levonogestrel	Janaud	(74)	France	32	26.1	–	6	Medium
Levonogestrel	Kauppinen-Mäkelin	(39)	Finland	15	–	–	3	Medium
Levonogestrel	Song	(40)	China	12	32.2	20.8	3	Medium
Levonogestrel	Kakis	(75)	Canada	8	22.4	22.1	24	Medium
Levonogestrel	Reisman	(76)	USA	155	26.8	25.2	4	Medium
Levonogestrel	Endrikat	(77)	Germany	23	22.7	22.2	13	Medium
Levonogestrel	Endrikat	(77)	Germany	25	24.2	22.5	13	Medium
Levonogestrel	Merki-Feld	(66)	Switzerland	8	20.3	–	3	Medium
Levonogestrel	Merki-Feld	(66)	Switzerland	8	22.0	–	3	Medium
Levonogestrel	Merki-Feld	(67)	Switzerland	6	20.8	24.2	3	High
Levonogestrel	Merki-Feld	(67)	Switzerland	6	22.8	21.6	3	High
Levonogestrel	Wiegratz	(52)	Germany	25	26.1	21.9	6	Low
Levonogestrel	Scharnagl	(78)	Austria	44	27.0	22.3	12	Low
Levonogestrel	Scharnagl	(78)	Austria	46	26.0	22.3	12	Low
Levonogestrel	Skouby	(79)	Denmark	22	23.5	21.1	12	Medium
Levonogestrel	Skouby	(79)	Denmark	27	24.1	21.9	12	Medium
Levonogestrel	Skouby	(80)	Denmark	9	22.0	–	6	Medium
Levonogestrel	Elkind-Hirsch	(81)	USA	20	29.5	29.5	6	Medium
Levonogestrel	Ågren	(82)	Finland	58	29.1	22.3	6	Medium
Levonogestrel	Junge	(53)	Germany	28	31.1	22.1	6	Medium
Levonogestrel	Beasley	(83)	USA	58	25.0	26.1	3	Low
Levonogestrel	Beasley	(83)	USA	51	24.6	26.7	3	Low
Levonogestrel	Shahnazi	(51)	Iran	69	28.9	28.5	3	Low
Norgestimate	Janaud	(74)	France	34	24.7	–	6	Medium
Norgestimate	Petersen	(64)	Denmark	17	23.5	20.3	6	Low
Norgestimate	Cibula	(84)	Czech Republic	14	23.2	22.1	6	Medium
Norgestimate	Essah	(85)	USA	10	–	32.6	3	Low
Norgestimate	Hagag	(23)	Israel	25	22.0	24.0	12	High

^a^ *n* refers to the number of women in the group receiving the progestin of interest.

In all but 2 of the included studies (one with dienogest and one with cyproterone), the estrogen present in the OC was ethinyl estradiol. Among the 143 intervention groups included, the progestin received was chlormadinone acetate in 2, cyproterone acetate in 27, desogestrel in 29, dienogest in 3, drospirenone in 17, gestodene in 17, levonorgestrel in 43 and norgestimate in 5.

**Figure 1:**
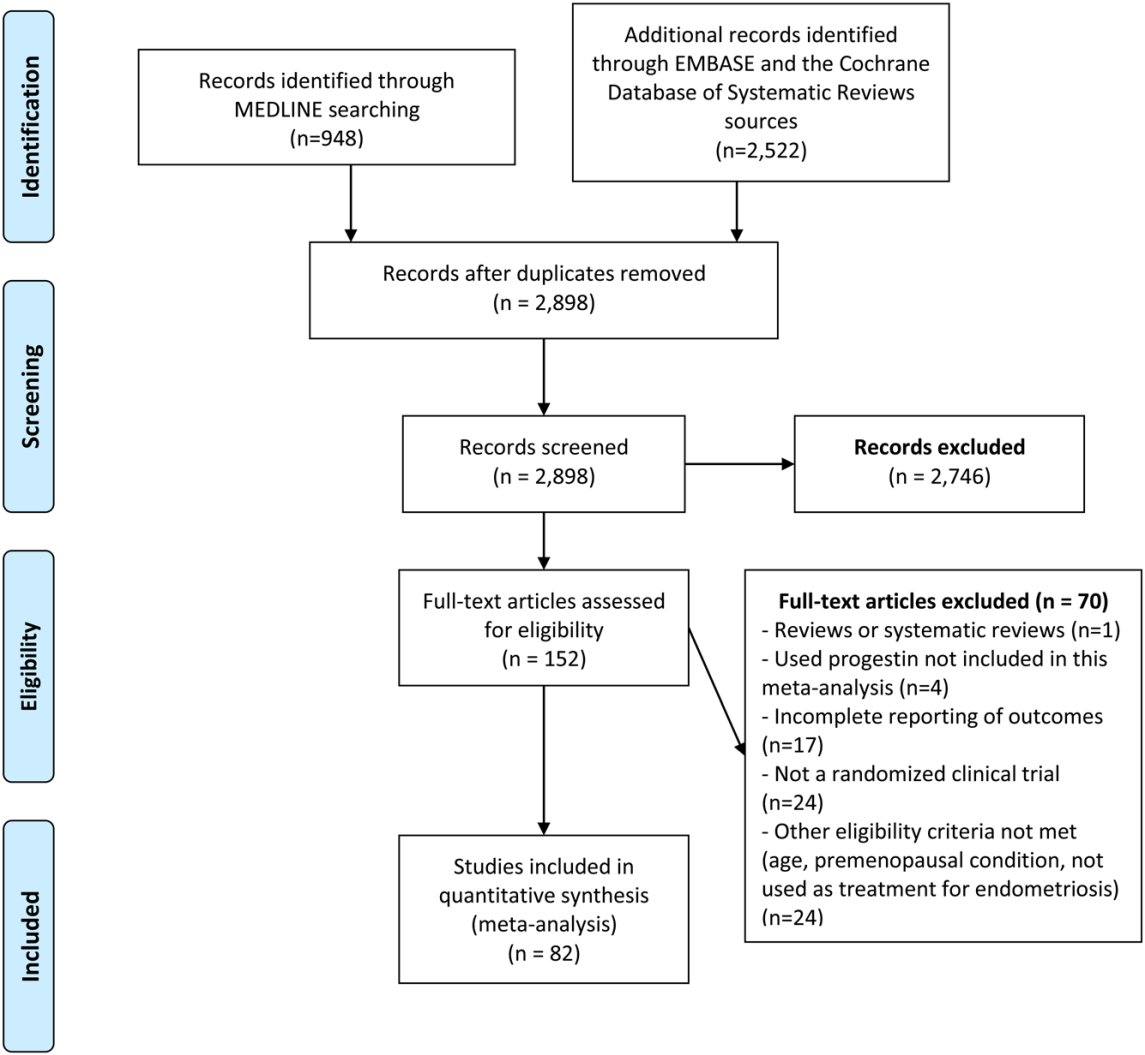
PRISMA flowchart of the study.

We screened 3470 references through database searching for articles published up to July 2020 (Fig. 1). The searches yielded 948 results from MEDLINE and 2522 from EMBASE and the Cochrane Database of Systematic Reviews. Based on this electronic search, we assessed for duplicates, discarded studies that clearly were not related to the interventions or outcomes of interest and downloaded the titles and abstracts of the remaining records. After abstract review, the number of potentially eligible studies was limited to 152. After procuring the full text of these studies, the complete information for baseline and follow-up measurements of study outcomes was evaluated by at least two independent authors. Seventy studies were excluded because they were reviews or systematic reviews, used a progestin not included in this meta-analysis, had incomplete information on outcomes, were not clinical trials, or failed to meet other eligibility criteria (Fig. 1).

### HDL cholesterol

**Figure 2:**
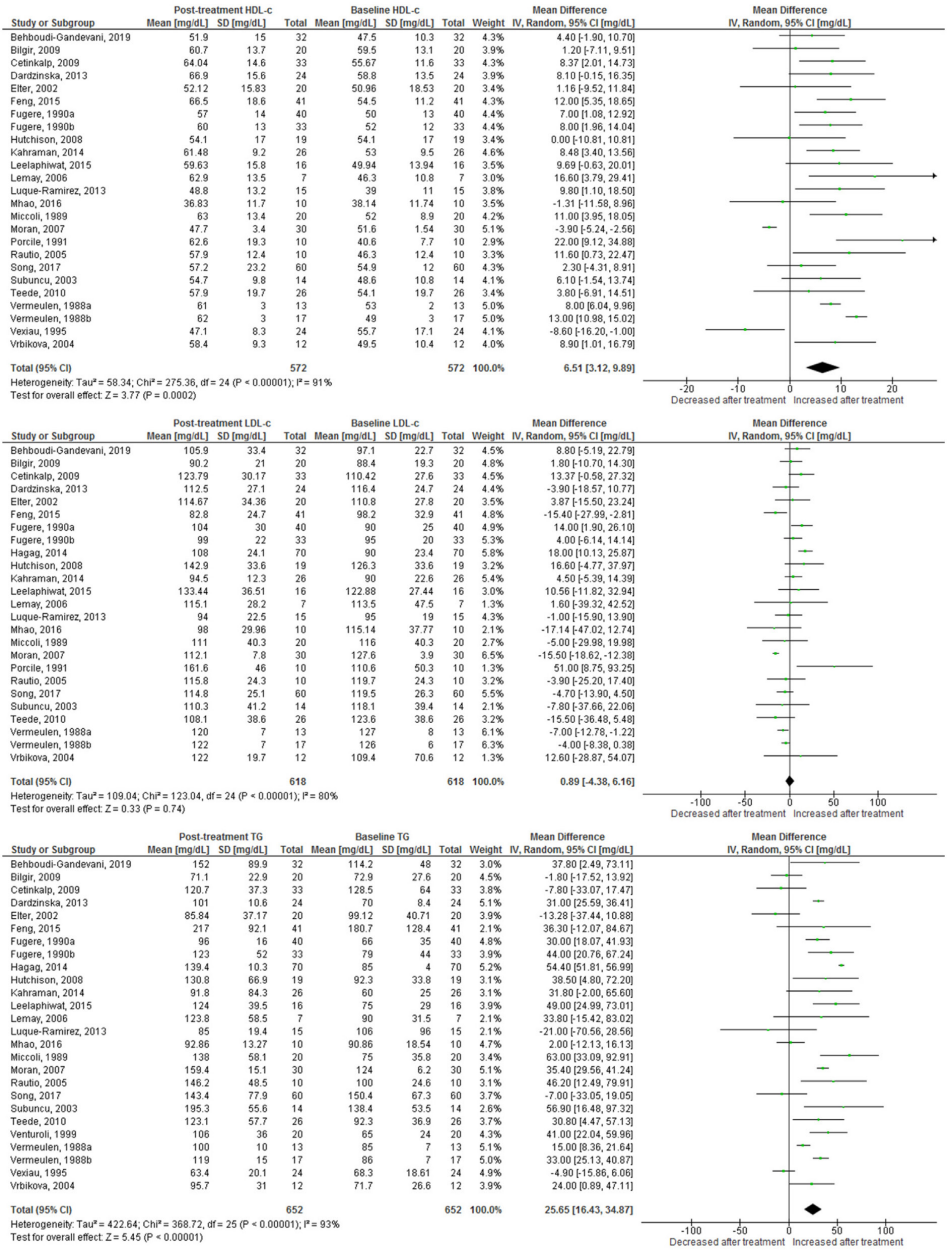
Changes in plasma HDL cholesterol, LDL cholesterol and triglycerides after use of cyproterone in clinical trials.

**Figure 3:**
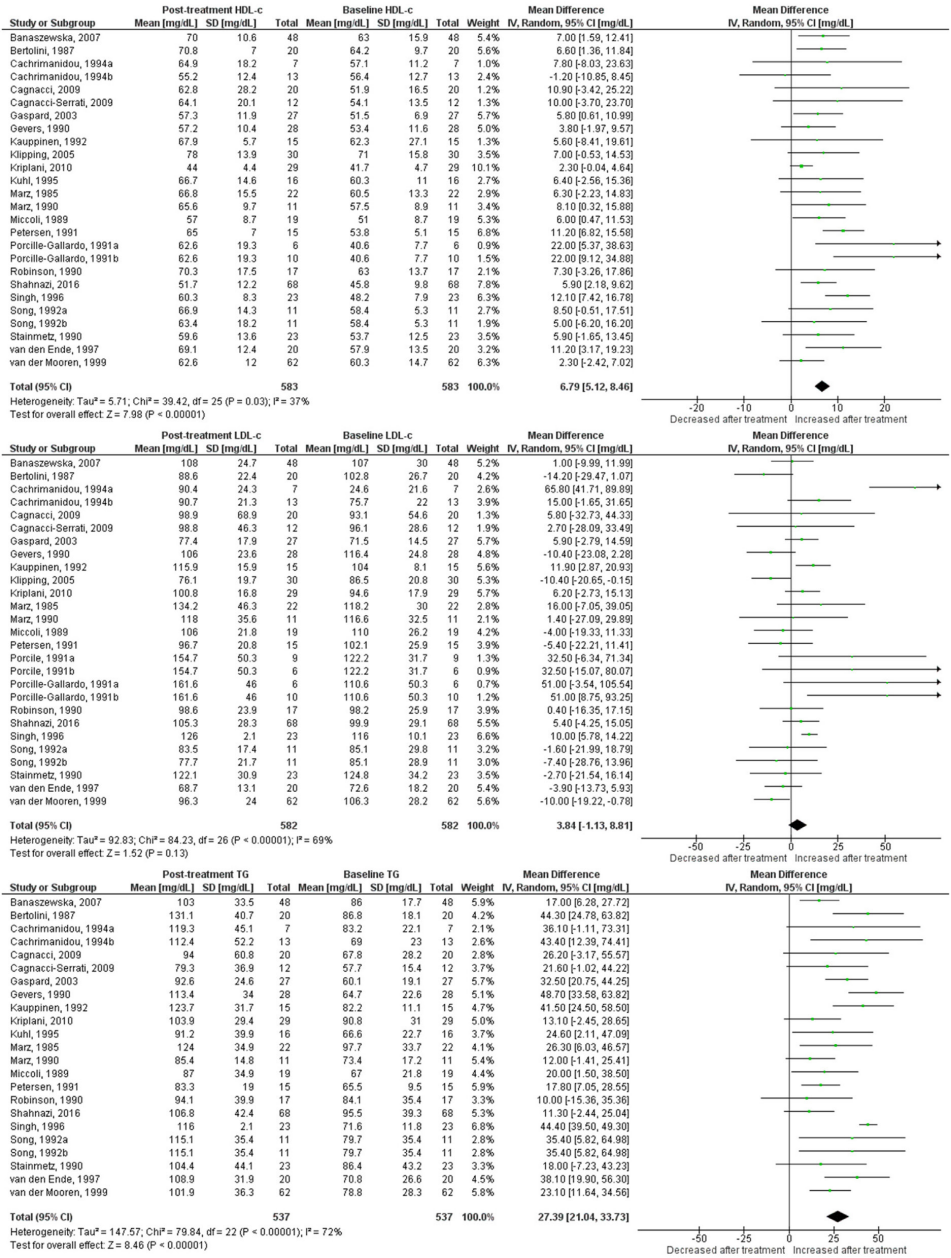
Changes in plasma HDL cholesterol, LDL cholesterol and triglycerides after use of desogestrel in clinical trials.

**Figure 4:**
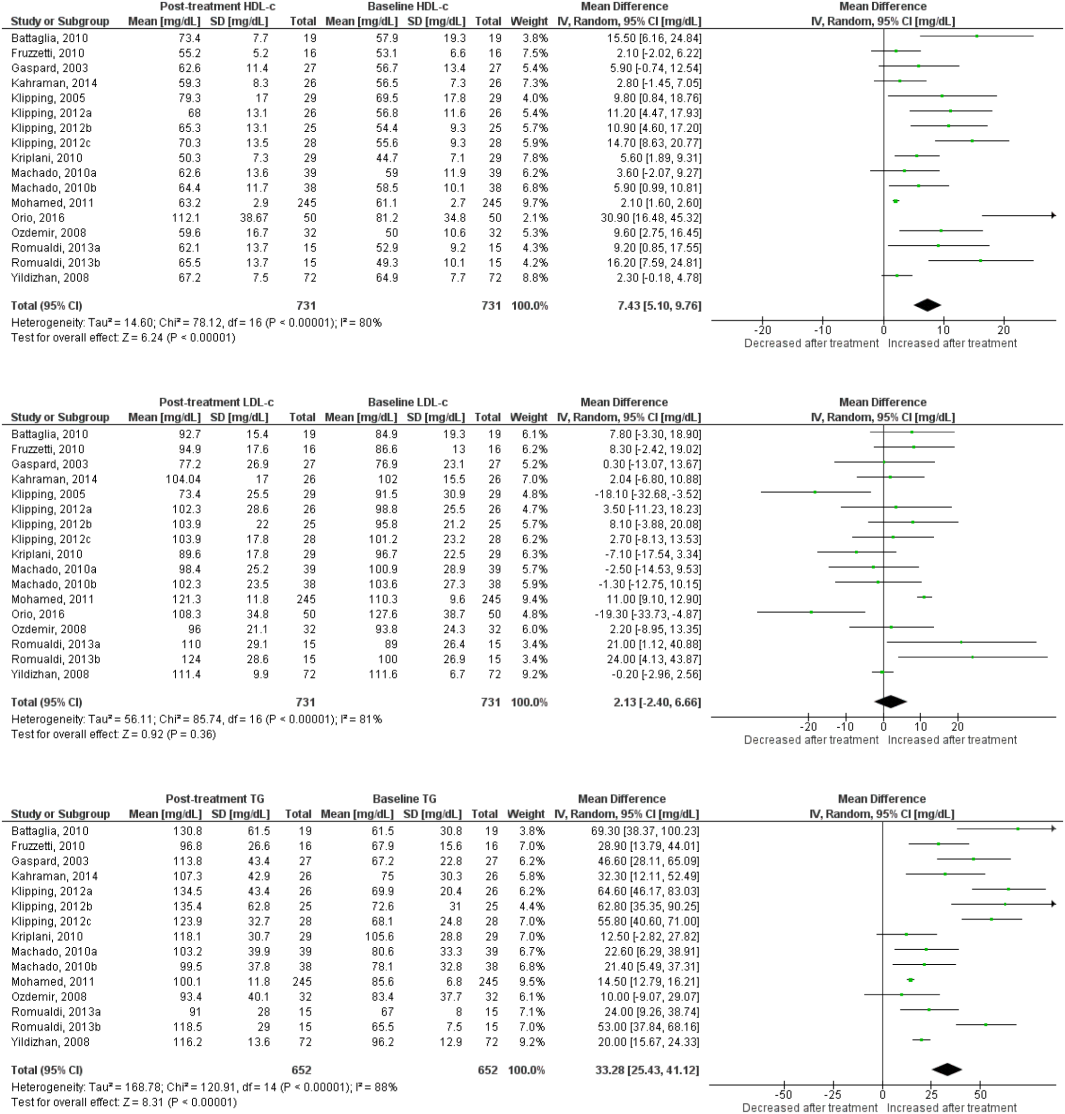
Changes in plasma HDL cholesterol, LDL cholesterol and triglycerides after use of drospirenone in clinical trials.

**Figure 5:**
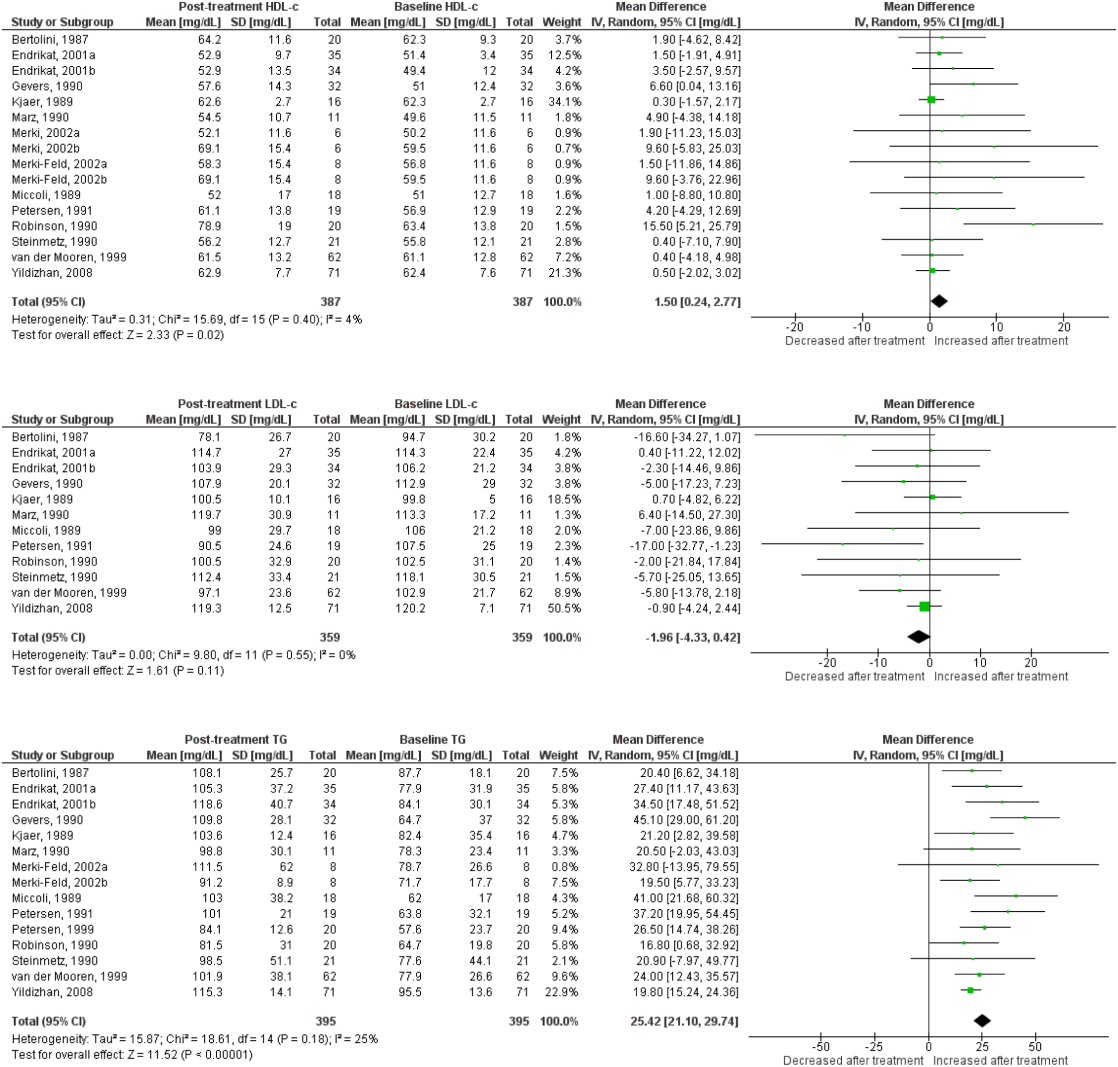
Changes in plasma HDL cholesterol, LDL cholesterol and triglycerides after use of gestodene in clinical trials.

**Figure 6:**
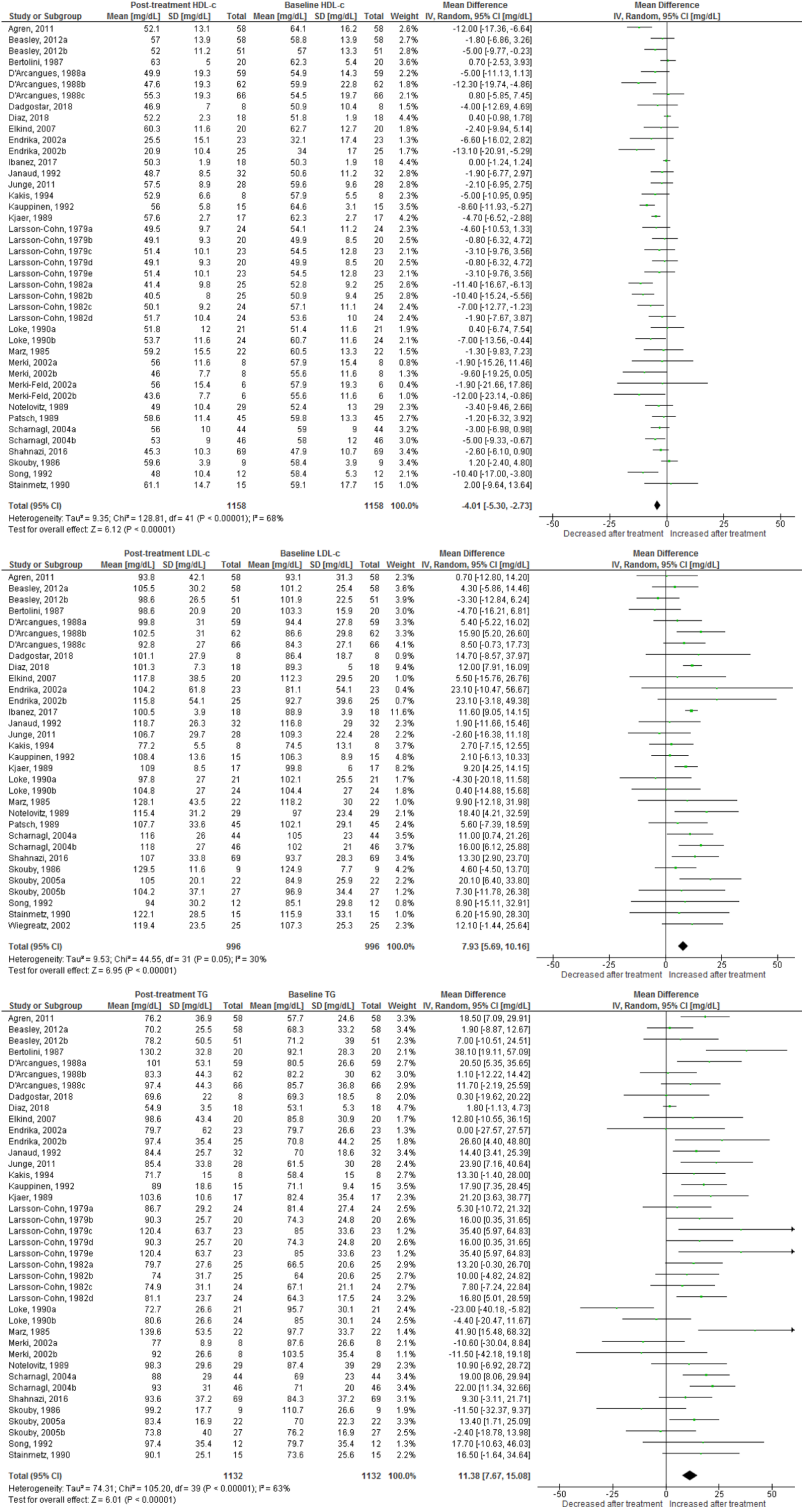
Changes in plasma HDL cholesterol, LDL cholesterol and triglycerides after use of levonorgestrel in clinical trials.

**Figure 7:**
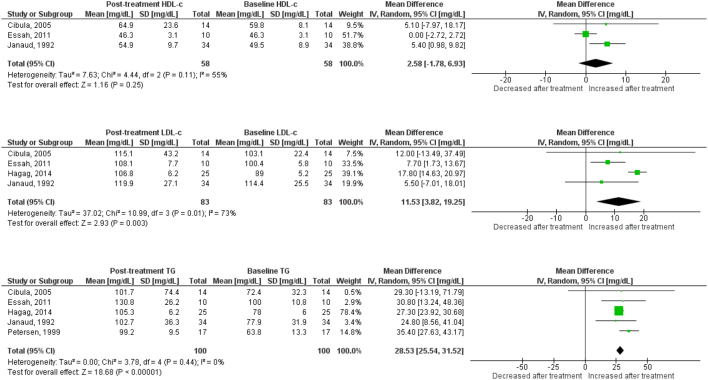
Changes in plasma HDL cholesterol, LDL cholesterol and triglycerides after use of norgestimate in clinical trials.

Use of OC containing chlormadinone (pMD: 9.6 mg/dL; 95% CI: 4.5 to 14.7) was associated with significant increases in HDLc. Similar results were found for cyproterone (pMD: 6.5 mg/dL; 95% CI: 3.1 to 9.9, Fig. 2), desogestrel (pMD: 6.8 mg/dL; 95% CI: 5.1 to 8.5, Fig. 3), drospirenone (pMD: 7.4 mg/dL; 95% CI: 5.1 to 9.8, Fig. 4) and to a lesser extent, gestodene (pMD: 1.5 mg/dL; 95% CI: 0.2 to 2.8) (Fig. 5). In the case of cyproterone, meta-regression analyses showed that older age was significantly correlated with smaller increases in HDLc (standardized beta= −0.58, *P* = 0.045). Contrastingly, levonorgestrel use decreased HDLc (pMD: −4.40 mg/dL; 95% CI: −5.67 to −3.13, Fig. 6). Studies of norgestimate that reported data on HDLc showed no significant changes (Fig. 7).

### LDL cholesterol

An increase in LDLc was observed for levonorgestrel (pMD: 6.8 mg/dL; 95% CI: 4.3 to 9.3), being significantly larger in long-term (>12 months) studies (pMD: 10.2 mg/dL; 95% CI: 6.2 to 14.2; *P* = 0.04 for interaction) (Table 2). Few studies of norgestimate reported LDLc, but these data showed a significant increase (pMD: 11.5 mg/dL; 95% CI: 3.8 to 19.3) (Fig. 7). LDLc increased significantly in women after cyproterone use only in long-term studies (pMD: 11.7 mg/dL; 95% CI: 3.3 to 20.1, *P* = 0.003 for interaction) (Table 2). Similarly, LDLc increased only with long-term use for desogestrel (pMD: 22.3 mg/dL; 95% CI: 5.7 to 38.9; *P* = 0.01 for interaction) and drospirenone (pMD: 6.3 mg/dL; 95% CI: 0.6 to 12.0; *P* = 0.04 for interaction) (Table 2). By contrast, use of dienogest-containing OC was associated with a significant reduction in LDLc (pMD: −7.7 mg/dL; 95% CI: −14.9 to −0.5).

**Table 2:** Changes in metabolic outcomes after use of oral contraceptives containing different progestins, by study duration.

Outcome	Subgroup (months)	N studies (participants)	pMD (95% CI)	*I*^2^	*P*-value for difference between subgroups	*P*-value for heterogeneity
Cyproterone						
BMI	<12	14 (300)	−0.13 (−0.45, 0.19)	0	0.35	0.66
	≥12	2 (50)	0.23 (−0.46, 0.92)	0		0.84
Glucose	<12	11 (233)	−1.63 (−3.11, −0.16)	11	0.64	0.34
	≥12	2 (50)	−3.11 (−9.09, 2.87)	97		<0.0001
TG	<12	18 (347)	23.7 (15.6, 31.9)	80	0.49	<0.0001
	≥12	6 (213)	32.6 (8.9, 56.2)	96		<0.0001
HDL	<12	18 (347)	6.87 (2.64, 11.1)	93	0.94	<0.0001
	≥12	4 (133)	6.55 (−0.56, 13.7)	81		0.0002
LDL	<12	18 (347)	−3.07 (−7.83, 1.69)	66	0.002	<0.0001
	≥12	5 (179)	11.7 (3.34, 20.2)	61		0.04
HOMA	<12	9 (216)	−0.32 (−0.85, 0.22)	97	0.64	<0.0001
	≥12	1 (26)	−0.55 (−1.39, 0.29)	NA		NA
Chlormadinone						
BMI	<12	1 (12)	0.20 (−2.45, 2.85)	NA	0.87	NA
	≥12	1 (20)	0.50 (−2.10, 3.10)	NA		NA
Glucose	<12	1 (12)	0 (−4.04, 5.04)	NA	0.28	NA
	≥12	1 (20)	−4.20 (−10.01, 1.61)	NA		NA
TG	<12	1 (12)	23.0 (5.80, 40.2)	NA	0.12	NA
	≥12	1 (20)	56.7 (17.6, 95.8)	NA		NA
HDL	<12	1 (12)	12.3 (1.01, 23.6)	NA	0.60	NA
	≥12	1 (20)	8.90 (3.22, 14.6)	NA		NA
LDL	<12	1 (12)	−7.70 (−32.9, 17.5)	NA	0.53	NA
	≥12	1 (20)	−0.76 (−13.7, 12.1)	NA		NA
HOMA	<12	–	NA	NA	–	NA
	≥12	–	NA	NA		NA
Desogestrel						
BMI	<12	6 (196)	0.11 (−0.45, 0.67)	0	0.16	0.79
	≥12	2 (15)	2.20 (−0.67, 5.07)	0		0.99
Glucose	<12	9 (281)	1.53 (−0.56, 3.61)	58	–	NA
	≥12	–	NA	NA		NA
TG	<12	18 (451)	25.9 (18.6, 33.2)	74	0.40	<0.0001
	≥12	4 (86)	33.1 (18.0, 48.2)	71		0.008
HDL	<12	19 (481)	6.69 (4.94, 8.45)	33	0.73	0.08
	≥12	7 (102)	7.63 (2.72, 12.5)	52		0.05
LDL	<12	18 (465)	0.19 (−4.37, 4.75)	58		0.001
	≥12	9 (117)	22.3 (5.66, 38.9)	80		<0.0001
HOMA	<12	–	NA	NA	–	NA
	≥12	–	NA	NA		NA
Drospirenone						
BMI	<12	5 (146)	−0.07 (−0.87, 0.73)	0	0.25	0.96
	≥12	4 (128)	−0.69 (−1.04, −0.16)	0		0.58
Glucose	<12	7 (223)	0.27 (−2.36, 2.90)	76	0.37	0.0003
	≥12	2 (271)	4.71 (−4.60, 14.01)	98		<0.0001
TG	<12	6 (173)	24.0 (12.4, 35.6)	61	0.06	0.03
	≥12	9 (479)	38.9 (28.4, 49.4)	92		<0.0001
HDL	<12	8 (252)	7.91 (4.11, 11.7)	67	0.78	0.003
	≥12	9 (479)	7.21 (4.12, 10.3)	82		<0.0001
LDL	<12	8 (252)	−2.96 (−9.71, 3.80)	62	0.04	0.01
	≥12	9 (479)	6.30 (0.56, 12.0)	84		<0.0001
HOMA	<12	5 (146)	−0.16 (−0.61, 0.28)	60	0.64	0.04
	≥12	6 (172)	0.10 (−0.91, 1.11)	NA		NA
Gestodene						
BMI	<12	2 (38)	0.56 (−0.50, 1.63)	0	0.47	0.64
	≥12	1 (71)	0.10 (−0.56, 0.76)	NA		NA
Glucose	<12	–	NA	NA	–	NA
	≥12	–	NA	NA		NA
TG	<12	12 (281)	25.7 (21.1, 30.3)	0	0.79	0.69
	≥12	3 (114)	28.0 (11.2, 44. 9)	77		0.01
HDL	<12	13 (273)	1.39 (−0.03, 2.81)	2	0.53	0.43
	≥12	3 (114)	2.80 (−1.39, 7.00)	42		0.18
LDL	<12	9 (245)	−3.24 (−6.88, 0.40)	2	0.37	0.42
	≥12	3 (359)	−1.01 (−4.20, 2.18)	0		0.64
HOMA	<12	–	NA	NA	–	NA
	≥12	–	NA	NA		NA
Levonorgestrel						
BMI	<12	–	NA	NA	–	NA
	≥12	–	NA	NA		NA
Glucose	<12	5 (199)	−3.0 (−11.1, 5.09)	87	0.35	<0.0001
	≥12	4 (296)	−8.16 (−15.3, −0.99)	88		<0.0001
TG	<12	25 (650)	13.7 (9.42, 17.9)	41	0.26	0.02
	≥12	13 (456)	9.02 (2.06, 16.0)	64		0.0008
HDL	<12	28 (707)	−4.19 (−5.73, −2.66)	58	0.59	<0.0001
	≥12	11 (407)	−4.94 (−7.18, −2.70)	31		0.15
LDL	<12	16 (496)	5.08 (2.42, 7.75)	0	0.04	0.49
	≥12	13 (456)	10.2 (6.15, 14.2)	21		0.23
HOMA	<12	–	NA	NA	–	NA
	≥12	–	NA	NA		NA
Norgestimate						
BMI	<12	–	NA	NA	–	NA
	≥12	–	NA	NA		NA
Glucose	<12	–	NA	NA	–	NA
	≥12	–	NA	NA		NA
TG	<12	5 (98)	36.3 (28.3, 44.2)	56	0.04	0.06
	≥12	1 (25)	27.3 (23.9, 30.7)	NA		NA
HDL	<12	–	NA	NA	–	NA
	≥12	–	NA	NA		NA
LDL	<12	4 (81)	9.02 (5.73, 12.3)	0	0.0002	0.86
	≥12	1 (25)	17.8 (14.6, 20.8)	NA		NA
HOMA	<12	–	NA	NA	–	NA
	≥12	–	NA	NA		NA

NA, not applicable, heterogeneity not calculable in this subgroup; NR, not reported.

### Plasma triglycerides

Most progestins induced a significant increase in plasma TG. The observed effect was largest for chlormadinone (pMD: 35.1 mg/dL; 95% CI: 3.4 to 66.8), followed by drospirenone (pMD: 33.3 mg/dL; 95% CI: 25.4 to 41.1), norgestimate (pMD: 28.5 mg/dL; 95% CI: 25.5 to 31.5), desogestrel (pMD: 27.4 mg/dL; 95% CI: 21.0 to 33.7), cyproterone (pMD: 25.6 mg/dL; 95% CI: 16.4 to 34.9), gestodene (pMD: 25.4 mg/dL; 95% CI: 21.1 to 29.7) and levonorgestrel (pMD: 12.1 mg/dL; 95% CI: 8.42 to 15.7) (Figs 2, 3, 4, 5 and 6). Only for norgestimate there was a difference between PCOS status subgroups, the impact on plasma TG was larger for studies in women without PCOS (*P* = 0.02 for interaction, Table 3).

**Table 3:** Changes in metabolic outcomes after use of oral contraceptives containing different progestins, in participants with or without polycystic ovary syndrome.

Outcome	Subgroup	N studies (participants)	pMD (95% CI)	*I*^2^	*P*-value for difference between subgroups	*P*-value for heterogeneity
Cyproterone						
BMI	PCOS	14 (306)	−0.06 (−0.37, 0.24)	0	0.92	0.63
	No PCOS	2 (44)	−0.11 (−1.01, 0.78)	0		0.50
Glucose	PCOS	13 (283)	−2.26 (−3.97, −0.56)	51	<0.0001	0.02
	No PCOS	1 (24)	−6.11 (−3.97, −0.56)	NA		NA
TG	PCOS	17 (393)	25.1 (13.8, 36.4)	92	0.70	<0.0001
	No PCOS	7 (167)	28.6 (15.1, 42.0)	88		<0.0001
HDL	PCOS	16 (323)	6.09 (1.91, 10.3)	84	0.49	<0.0001
	No PCOS	7 (157)	8.17 (3.95, 12.4)	85		<0.0001
LDL	PCOS	17 (393)	0.08 (−8.01, 8.16)	83	0.80	<0.0001
	No PCOS	6 (133)	1.49 (−6.23, 9.22)	72		0.003
Chlormadinone						
BMI	PCOS	1 (20)	0.50 (−2.10, 3.10)	NA	0.87	NA
	No PCOS	1 (12)	0.20 (−2.45, 2.85)	NA		NA
Glucose	PCOS	1 (20)	−4.20 (−10.01, 1.61)	NA	0.28	NA
	No PCOS	1 (12)	0 (−4.04, 5.04)	NA		NA
TG	PCOS	1 (20)	56.7 (17.6, 95.8)	NA	0.12	NA
	No PCOS	1 (12)	23.0 (5.80, 40.2)	NA		NA
HDL	PCOS	1 (20)	8.90 (3.22, 14.6)	NA	0.60	NA
	No PCOS	1 (12)	12.3 (1.01, 23.6)	NA		NA
LDL	PCOS	1 (20)	1.70 (−13.3, 16.7)	NA	0.53	NA
	No PCOS	1 (12)	−7.70 (−32.9, 17.5)	NA		NA
Desogestrel						
BMI	PCOS	2 (77)	0.48 (−0.82, 1.78)	41	0.64	0.19
	No PCOS	6 (134)	0.12 (−0.57, 0.82)	0		0.78
Glucose	PCOS	2 (77)	3.84 (−2.58, 9.54)	76	0.45	0.04
	No PCOS	7 (204)	0.99 (−1.30, 3.29)	55		0.04
TG	PCOS	2 (77)	15.7 (6.92, 24.6)	0	0.02	0.69
	No PCOS	21 (460)	28.9 (22.3, 35.4)	70		<0.0001
HDL	PCOS	2 (77)	3.99 (−0.43, 8.41)	59	0.17	0.12
	No PCOS	24 (506)	7.26 (5.68, 8.83)	14		0.27
LDL	PCOS	2 (77)	4.13 (−2.80, 11.1)	0	0.99	0.47
	No PCOS	25 (505)	4.09 (−1.47, 9.65)	71		<0.0001
Drospirenone						
BMI	PCOS	8 (202)	−0.04 (−0.68, 0.61)	0	0.02	0.99
	No PCOS	9 (274)	−0.60 (−1.04, −0.16)	0		0.58
Glucose	PCOS	6 (172)	0.12 (−2.63, 2.88)	80	0.33	0.0001
	No PCOS	3 (322)	3.55 (−2.76, 9.85)	95		<0.0001
TG	PCOS	7 (152)	32.0 (17.5, 44.6)	76	0.64	0.0003
	No PCOS	8 (500)	35.1 (24.9, 45.2)	91		<0.0001
HDL	PCOS	8 (202)	9.26 (4.84, 13.7)	75	0.30	0.0002
	No PCOS	9 (529)	6.46 (3.65, 9.28)	78		<0.0001
LDL	PCOS	8 (202)	3.44 (−4.29, 11.2)	68	0.99	0.003
	No PCOS	9 (529)	1.30 (−4.70, 7.30)	87		<0.0001
Norgestimate						
BMI	PCOS	2 (24)	6.14 (−1.49, 1.77)	0	0.95	0.69
	No PCOS	1 (17)	0.20 (−0.75, 1.15)	NA		NA
Glucose	PCOS	2 (24)	−0.16 (−2.45, 2.13)	0	0.30	0.50
	No PCOS	1 (17)	1.80 (−1.09, 4.69)	NA		NA
TG	PCOS	3 (49)	27.4 (24.1, 30.8)	0	0.06	0.93
	No PCOS	3 (74)	37.1 (27.5, 46.7)	74		0.02
HDL	PCOS	2 (24)	0.21 (−2.45, 2.87)	0	0.02	0.45
	No PCOS	2 (57)	8.70 (2.14, 15.3)	76		0.04
LDL	PCOS	3 (49)	13.0 (4.37, 21.7)	77	0.47	0.01
	No PCOS	2 (57)	9.54 (5.55, 13.5)	0		0.50

NA, not applicable, heterogeneity not calculable in this subgroup; NR, not reported.

### BMI

Drospirenone caused a small reduction in BMI (pMD: –0.60 kg/m^2^; 95% CI: –1.04 to –0.16). However, this effect was driven by a study among women without PCOS ((65), pMD: –1.1 kg/m^2^; 95% CI: –1.7 to –0.5; *P* = 0.02 for interaction; Table 3). None of the other progestins included in this study had a significant impact on BMI (Figs 2, 3, 5, 6 and 7).

### Insulin resistance

None of the studied progestins had a significant impact on HOMA-IR.

### Plasma glucose

Women taking cyproterone showed a trivial yet statistically significant decrease in fasting plasma glucose (FPG) (pMD: –2.7 mg/dL; 95% CI: –4.8 to –0.7). This reduction was significantly larger in the only cyproterone study in participants without PCOS than in the studies among women with PCOS (*P* = 0.02 for interaction; Table 3). None of the other progestins showed a significant effect on FPG.

### Risk of bias

Out of the 82 studies included, 32 were found to be of low ROB, 40 of medium ROB and 10 of high ROB (Table 1). We performed a sensitivity analysis in which we removed the 10 studies with high ROB and repeated meta-analyses for all OCs and all metabolic outcomes (Supplementary Table 2). Despite minor numerical differences, the significance of modifications in metabolic parameters and the central results of the study remained the same.

### Publication bias analyses

Visual examination of funnel plots and formal testing for publication bias with Egger’s and Begg’s tests revealed no indication of publication bias for most outcomes in most progestins (Supplementary Figs 1, 2, 3, 4 and 5). Four intervention-outcome pairs showed indication of publication bias: LDLc change after desogestrel, TG change after drospirenone, HDLc change after drospirenone and LDLc change after levonorgestrel. There was a study in which there was a 63 mg/dL increase in LDLc after use of a desogestrel-containing OC (51), this outlier contributed only 2.6% of the weighted mean difference but was largely responsible for an asymmetric funnel plot and significant Egger and Begg’s tests (Supplementary Fig. 2). Change in TG after drospirenone also exhibited an asymmetric funnel plot and significant Egger and Begg’s tests (Supplementary Fig. 3), but asymmetry was mainly due to small studies reporting larger increases in TG. After trim-and-fill analysis for these outcomes (Supplementary Fig. 6), only the modification of HDLc after drospirenone use was affected by publication bias adjustment, the increase in HDLc became smaller and borderline significant.

## Discussion

In this systematic review and meta-analysis, we estimated the effect of OCs containing different progestins on plasma lipids and other variables related to metabolic health, using data from randomized trials, including premenopausal women. The lipid fractions most influenced by OCs were plasma triglycerides and HDLc. All progestins except dienogest (whose studies did not report changes in TG) induced a significant increase in plasma TG, which ranged numerically between 12.1 mg/dL (levonorgestrel) and 35.1 mg/dL (chlormadinone), consistent with the existing literature from nonrandomized studies.

Most of the newer progestins including chlormadinone, cyproterone, desogestrel and drospirenone induced statistically and clinically significant increases in HDLc, as had been found in previous studies in multiple different populations (88). Even though in observational studies gestodene induced significant increases in HDLc (2), our study showed a numerically marginal increase. Meanwhile, norgestimate did not affect HDLc and levonorgestrel significantly reduced it. Clinical trials of dienogest did not report on HDLc but a previous observational study of patients with endometriosis did show a tendency to lower it (89). On the other hand, the effect of OCs on plasma LDLc was generally more modest, and affected to the greater extent by the duration of use. The above-mentioned observational study of dienogest in endometriosis found no effect of dienogest on lipid profile variables (89), in contrast with our meta-analysis, which showed a 7 mg/dL decrease in LDLc. In accordance with earlier literature (3), levonorgestrel and norgestimate relevantly increased LDLc (7 and 11 mg/dL, respectively) (Table 4). For progestins with an effect on LDLc, this effect was evident mostly for studies of long-term (>12 months) use. A recent randomized cross-over trial that compared OCs containing newer progestins vs levonorgestrel in women with PCOS found the largest positive impact for drospirenone, both in terms of anti-androgenic activity (reduction in free androgen index and acne) and modification of plasma lipids (reduction of LDLc and increase of HDLc) (90).

**Table 4:** Summary of changes in metabolic outcomes after use of oral contraceptives containing different progestins in randomized clinical trials.

Progestin in OC	Metabolic outcome					
	LDLc	HDLc	TG	BMI	FPG	HOMA-IR
Chlormadinone	↔	↑	↑	↔	↔	NA
Cyproterone	↔	↑	↑	↔	↓	↔
Desogestrel	↔	↑	↑	↔	↔	↔
Dienogest	↓	NA	NA	NA	NA	NA
Drospirenone	↔	↑	↑	↓	↔	↔
Gestodene	↔	↔↑	↑	↔	NA	NA
Levonorgestrel	↑	↓	↑	↔	↔	↔
Norgestimate	↑	↔	↑	↔	↔	NA

FPG, fasting plasma glucose; HDLc, HDL cholesterol; HOMA-IR, homeostasis model assessment-insulin resistance; LDLc, LDL cholesterol; NA, not available; TG, triglycerides.

Perhaps unexpectedly, we found a negligible or non-existent effect of OCs on body weight. Drospirenone use was accompanied by a significant yet small 0.6 kg/m^2^ reduction in BMI, an effect that was not significant in prior individual observational studies or short-term trials (91). None of the other progestins affected BMI. Though few clinical trials were available, our analyses revealed that cyproterone use was associated with a slight decrease in plasma glucose. HOMA-IR, an index of insulin resistance in the fasting state, was not affected by any of the progestins included in our analyses.

Interestingly, BMI did not appear to be a significant modifier of the metabolic effects for most of the studied OCs, and age was a significant modifier for only one of the outcomes (HDLc change after cyproterone). Thus, the intrinsic pharmacology of each OC seemed to be a greater determinant of its metabolic effect than its interaction with the patient’s age or BMI.

A recent meta-analysis compared the effects of OCs on metabolic parameters, specifically in women with PCOS patients based on the type of progestin and duration of follow-up (88). Similar to our results, the most salient modifications in plasma lipids were increases in both triglycerides and HDLc, with elevations of HDLc occurring sooner than those of TG for most agents. This study also found a significant increase in LDL with use for 12 months or longer for all progestins, something that we also found for levonorgestrel, desogestrel, drospirenone and cyproterone; but not for dienogest, which in fact reduced LDLc. Also, in line with our results, this prior systematic review in PCOS found no impact of OCs on body weight or plasma glucose.

We observed that the selection criteria for inclusion in this review resulted in a sample of studies with a reasonably low risk of bias. Most (72/82, or 87.8%) of the studies included were of low or medium risk of bias, and the exclusion of the remaining 10 studies did not modify the magnitude or significance of the modifications in metabolic parameters estimated in the complete sample.

The main strengths of our study are the inclusion of clinical trials only, the analysis of OCs containing multiple different progestins and the study of various relevant metabolic outcomes. The fact that we restricted our search to studies of premenopausal women receiving OCs at contraceptive doses (as opposed to the doses used for hormonal replacement or for the treatment of endometriosis), may also have contributed to a reduced clinical heterogeneity of the studies. The meta-analysis addresses a relevant question and its results provide useful guidance for clinicians.

The main limitations of our study include the presence of a fair amount of heterogeneity for most outcomes, and the scarcity of high-quality clinical trials reporting on variables related to carbohydrate metabolism like glycemia and HOMA-IR. In order to take into account the very likely existence of between-studies heterogeneity on the observed effects, we synthesized study results using random-effects models for all outcomes. Concerning the effects of OC’s on glycemia and HOMA-IR, it is important for these parameters to be included in future studies of OCs, in order to perform a more complete assessment of the physiological impact of hormonal contraception.

## Conclusion

OCs containing different progestins have small, but potentially clinically important effects on the metabolic profile in premenopausal women. Most progestins increase plasma TG and some raise HDLc. In general, OCs have minor or no effects on LDLc, BMI, HOMA-IR and FPG. Baseline lipid and glucose testing should be considered to help determine the most appropriate OC prescription for women.

## Supplementary Material

Supplementary Table 1. Search strategy.

Supplemental Table 2. Changes in metabolic outcomes after use of oral contraceptives containing different progestins, before or after removing studies with high risk of bias (ROB) (n=10 studies).

Supplemental Figure 1. Funnel plots of mean weighted difference versus standard error of the weighted mean difference for metabolic outcomes in studies of oral contraceptives containing cyproterone. Egger´s and Begg´s tests were only calculated for outcomes in which there were at least 5 observations (studies).

Supplemental Figure 2. Funnel plots of mean weighted difference versus standard error of the weighted mean difference for metabolic outcomes in studies of oral contraceptives containing desogestrel. Egger´s and Begg´s tests were only calculated for outcomes in which there were at least 5 observations (studies).

Supplemental Figure 3. Funnel plots of mean weighted difference versus standard error of the weighted mean difference for metabolic outcomes in studies of oral contraceptives containing drospirenone. Egger´s and Begg´s tests were only calculated for outcomes in which there were at least 5 observations (studies).

Supplemental Figure 4. Funnel plots of mean weighted difference versus standard error of the weighted mean difference for metabolic outcomes in studies of oral contraceptives containing gestodene. Egger´s and Begg´s tests were only calculated for outcomes in which there were at least 5 observations (studies).

Supplemental Figure 5. Funnel plots of mean weighted difference versus standard error of the weighted mean difference for metabolic outcomes in studies of oral contraceptives containing levonorgestrel. Egger´s and Begg´s tests were only calculated for outcomes in which there were at least 5 observations (studies).

Supplemental Figure 6. Trim-and-fill analysis of the four intervention-outcome pairs that showed indication of publication bias. Upper-left: Desogestrel and LDLc, upper-right: Drospirenone and TG, lower-left: Drospirenone and HDLc, lower-right: Levonorgestrel and LDLc. Only the modification of HDLc after drospirenone use was affected by publication bias adjustment.

Supplemental Figure 7. PRISMA checklist of the study.

## Declaration of interest

R J de Souza has served as an external resource person to the World Health Organization’s Nutrition Guidelines Advisory Group on tra fats, saturated fats, and polyunsaturated fats. The WHO paid for his travel and accommodation to attend meetings from 2012 to 2017 to present and discuss this work. He has also done contract research for the Canadian Institutes of Health Research’s Institute of Nutrition, Metabolism, and Diabetes, Health Canada, and the World Health Organization for which he received remuneration. He has received speaker’s fees from the University of Toronto, and McMaster Children’s Hospital. He has held grants from the Canadian Foundation for Dietetic Research, Population Health Research Institute, and Hamilton Health Sciences Corporation as a principal investigator, and is a co-investigator on several funded team grants from Canadian Institutes of Health Research. He serves as an independent director of the Helderleigh Foundation (Canada).

## Funding

Support for this study came from the Office of the Vice Provost for Research (Vicerrectoría de Investigaciones), School of Medicine, Universidad de los Andes in Bogotá, Colombia. C O Mendivil has received speaker honoraria or has participated in advisory boards for Abbott Laboratories, Amgen, AstraZeneca, Beckman Coulter, Bristol-Myers-Squibb, Boehringer Ingelheim, Café de Colombia, Colmédica, Craveri, Jannsen, LabCare Colombia, Merck S A, Novamed, Novartis, Novo Nordisk, Pfizer, PTC Therapeutics, Rochem Biocare, Sanofi and Sicma Pharma. He has been a consultant to Alpina S A, Bavaria S A, Cuquerella Consulting and Team Foods Colombia.
