# Supplementary material for: Effects of oral contraceptives on metabolic parameters in adult premenopausal women: a meta-analysis
Source: Endocr Connect. 2020 Sep 10;9(10):978–98. doi: 10.1530/EC-20-0423 (PMC7576645; doi:10.1530/EC-20-0423)
Supplement: Supplementary Table 1. Search strategy. [file supplementary_table_1.pdf]

**Supplementary Table 1.** Search strategy.	
**Embase**	**Medline**
1. oral contraceptive agent/	1. Contraceptives, Oral, Combined/
2. cyproterone/	2. Cyproterone/
3. cyproterone acetate plus ethinylestradiol/	3. Desogestrel/
4. desogestrel/	4. Chlormadinone Acetate/
5. desogestrel plus ethinylestradiol/	5. dienogest.nm.
6. chlormadinone acetate/	6. drospirenone.nm.
7. dienogest/	7. gestodene.nm.
8. drospirenone/	8. Levonorgestrel/
9. gestodene/	9. nomegestrol acetate.nm.
10. levonorgestrel/	10. norgestimate.nm.
11. nomegestrol acetate/	11. or/1-10
12. norgestimate/	12. norgestimate.mp.
13. or/1-12	13. nomegestrol.mp.
14. norgestimate.mp.	14. levonorgestrel.mp.
15. nomegestrol.mp.	15. gestodene.mp.
16. levonorgestrel.mp.	16. drospirenone.mp.
17. gestodene.mp.	17. dienogest.mp.
18. drospirenone.mp.	18. desogestrel.mp.
19. dienogest.mp.	19. chlormadinone.mp.
20. desogestrel.mp.	20. cyproterone.mp.
21. chlormadinone.mp.	21. or/12-20
22. cyproterone.mp.	22. 11 or 21
23. or/14-22	23. cholesterol, ldl/
24. low density lipoprotein cholesterol/	24. triglycerides/
25. high density lipoprotein cholesterol/	25. cholesterol, hdl/
26. triacylglycerol/	26. insulin resistance/
27. insulin resistance/	27. body mass index/
28. body mass/	28. blood glucose/
29. glucose blood level/	29. or/23-28
30. or/24-29	30. ldl cholesterol.mp.
31. ldl cholesterol*.mp.	31. triglyceride*.mp.
32. tri-glycerides.mp.	32. tri-glyceride*.mp.
33. triglycerides.mp.	33. hdl cholesterol*.mp.
34. hdl cholesterol*.mp.	34. homa-IR.mp.
35. homa-IR.mp.	35. body-mass ind*.mp.
36. body mass ind*.mp.	36. fasting plasma glucose.mp.
37. fasting plasma glucose.mp.	37. or/30-35
38. or/31-37	38. lipids/
39. lipid/	39. lipid.mp.
40. lipid.mp.	40. lipids.mp.
41. lipids.mp.	41. lipoproteins/
42. lipoprotein/	42. lipoproteins, IDL/
43. low density lipoprotein/	43. lipoproteinsn, LDL/
44. intermediate density lipoprotein/	44. lipoproteins, HDL/
45. high density lipoprotein/	45. lipoproteins, VLDL/
46. very low density lipoprotein/	46. lipoprotein*.mp.
47. lipoprotein*.mp.	47. triacetin.mp.
48. triacetin/	48. triolein.mp.
49. triacetin.mp.	49. triacetin/
50. triolein/	50. triolein/
51. triolein.mp.	51. triacylglycerol*.mp.

52. triacylglycerol*.mp.	52. (HDL or LDL or VLDL or IDL or TG or TAG).mp.
53. (HDL or LDL or IDL or VLDL or TG or TAG).mp.	53. cholesterol*.mp.
54. cholesterol*.mp.	54. apolipoproteins a/
55. apolipoprotein/	55. apolipoproteins a-i/
56. apolipoprotein A/	56. (apo* a1 or apo*a 1 or apo* a i or apo* ai).mp.
57. apolipoprotein A1/	57. (proapoliprotein adj1 (ai or a1 or a i or a-1)).mp.
58. apolipoprotein B/	58. apoliproteins B/
59. apolipoprotein B100/	59. apo* b.mp.
60. apolipoprotein B48/	60. exp waist circumference/
61. (apo* a1 or apo*a 1 or apo* a i or apo* ai).mp.	61. (waist adj3 (circumference* or ratio*)).mp.
62. (proapoliprotein adj1 (ai or a1 or a i or a-1)).mp.	62. glucose/
63. waist circumference/	63. glucose.mp.
64. (waist adj3 (circumference* or ratio*)).mp.	64. blood pressure*.mp.
65. glucose/	65. BMI.mp.
66. glucose*.mp.	66. glucose intolerance/
67. blood pressure/	67. exp insulin resistance/
68. blood pressure*.mp.	68. (insulin adj2 (resistance or sensitivity)).mp.
69. BMI.mp.	69. 29 or 37
70. glucose intolerance/	70. or/38-68
71. insulin resistance/	71. controlled clinical trial.pt.
72. (insulin adj2 (resistance or sensitivity)).mp.	72. randomized controlled trial.pt.
73. or/39-72	73. random*.mp.
74. randomized controlled trial/	74. rct*.mp.
75. "controlled clinical trial (topic)"/ or exp "clinical trial (topic)"/	75. Random Allocation/
76. randomization/	76. randomized controlled trials as topic/
77. random*.mp.	77. clinical trial*.mp.
78. rct*.mp.	78. clinical trial*.pt.
79. exp clinical trial/	79. exp Clinical Trials as Topic/
80. ((singl* or doubl* or tripl* or trebl*) adj3 (blind* or mask*)).mp.	80. exp clinical trial/
81. "systematic review"/	81. ((singl* or doubl* or tripl* or trebl*) adj3 (blind* or mask*)).mp.
82. systematic review*.mp.	82. systematic review*.mp.
83. meta analysis/	83. meta analys?s.mp.
84. meta analys?s.mp.	84. or/71-83
85. or/74-84	85. or/23-69
86. (13 or 23) and (30 or 38 or 73) and 85	86. 22 and 84 and 85
87. limit 86 to (english or german or portuguese or spanish)	87. limit 86 to (english or german or portuguese or spanish)
88. animals/ not (humans/ and animals/)	88. animals/ not (humans/ and animals/)
89. 87 not 88	89. 87 not 88
90. remove duplicates from 89	90. remove duplicates from 89
