# Supplementary material for: Effects of oral contraceptives on metabolic parameters in adult premenopausal women: a meta-analysis
Source: Endocr Connect. 2020 Sep 10;9(10):978–98. doi: 10.1530/EC-20-0423 (PMC7576645; doi:10.1530/EC-20-0423)
Supplement: Supplemental Table 2. Changes in metabolic outcomes after use of oral contraceptives containing different progestins, before or after removing studies with high risk of bias (ROB) (n=10 studies). [file supplementary_table_2.pdf]

Supplemental Table 2. Changes in metabolic outcomes after use of oral contraceptives containing different progestins, before or after removing studies with high risk of bias (ROB) (n=10 studies).

**Progestin**	**Outcome**	**N studies (participants), complete study sample**	**pMD (95%CI), complete study sample**	**N studies (participants), excluding high ROB studies**	**pMD (95%CI), excluding high ROB studies**
Cyproterone	BMI	16 (350)	0.07 (-0.36, 0.22)	14 (307)	-0.10 (-0.39, 0.20)
	Glucose	13 (283)	-2.74 (-4.81, -0.67)	12 (267)	-3.39 (-5.72, -1.07)
	TG	24 (560)	25.7 (14.4, 34.9)	20 (440)	26.9 (19.2, 34.6)
	HDL	22 (480)	6.51 (3.12, 9.9)	20 (430)	6.72 (2.81, 10.6)
	LDL	23 (527)	0.89 (-4.38, 6.16)	19 (406)	-1.11 (-6.42, 4.21)
	HOMA	10 (242)	-0.31 (-0.76, 0.15)	7 (202)	-0.19 (-0.48, 0.10)
Chlormadinone	BMI	2 (32)	0.35 (-1.5, 2.21)	2 (32)	0.35 (-1.5, 2.21)
	Glucose	2 (32)	-1.84 (-5.93, 2.24)	2 (32)	-1.84 (-5.93, 2.24)
	TG	2 (32)	35.1 (3.41, 66.8)	2 (32)	35.1 (3.41, 66.8)
	HDL	2 (32)	9.6 (4.51, 14.7)	2 (32)	9.6 (4.51, 14.7)
	LDL	2 (32)	-0.76 (-13.7, 12.1)	2 (32)	-0.76 (-13.7, 12.1)
	HOMA	-	-	-	-
Desogestrel	BMI	8 (211)	0.19 (-0.36, 0.73)	8 (211)	0.19 (-0.36, 0.73)
	Glucose	9 (281)	1.53 (-0.56, 3.61)	9 (281)	1.53 (-0.56, 3.61)
	TG	22 (537)	27.4 (21.0-33.7)	21 (517)	26.8 (20.2, 33.3)
	HDL	26 (583)	6.79 (5.12, 8.46)	24 (563)	6.08 (4.90, 7.26)
	LDL	27 (582)	3.84 (-1.13, 8.81)	25 (562)	1.38 (-2.98, 5.74)
	HOMA	2 (91)	0.34 (0.0, 0.69)	2 (91)	0.34 (0.0, 0.69)
Drospirenone	BMI	9 (274)	-0.60 (-1.04, -0.16)	8 (202)	-0.04 (-0.68, 0.61)
	Glucose	9 (494)	1.35 (-1.59, 4.29)	9 (494)	1.35 (-1.59, 4.29)
	TG	15 (652)	33.3 (25.4, 41.1)	14 (580)	35.5 (24.7, 46.3)
	HDL	17 (731)	7.43 (5.1, 9.76)	16 (659)	8.17 (5.46, 10.9)
	LDL	17 (731)	6.15 (4.75, 7.55)	16 (659)	8.36 (6.73, 9.98)
	HOMA	6 (172)	-0.14 (-0.54, 0.26)	6 (172)	-0.14 (-0.54, 0.26)
Gestodene	BMI	3 (109)	0.23 (-0.33, 0.79)	3 (109)	0.23 (-0.33, 0.79)
	Glucose	2 (82)	1.64 (-0.94, 4.22)	2 (82)	1.64 (-0.94, 4.22)
	TG	15 (395)	25.4 (21.1, 29.7)	14 (387)	25.5 (21.0, 30.0)
	HDL	16 (387)	1.50 (0.24, 2.77)	15 (379)	1.68 (0.28, 3.07)
	LDL	12 (604)	-1.96 (-4.33, 0.42)	12 (604)	-1.96 (-4.33, 0.42)
	HOMA	-	-	-	-
Levonorgestrel	BMI	5 (270)	0.12 (-0.29, 0.53)	5 (270)	0.12 (-0.29, 0.53)
	Glucose	11 (322)	-4.68 (-8.81, -0.55)	11 (322)	-4.68 (-8.81, -0.55)
	TG	38 (1106)	11.4 (7.67, 15.1)	36 (1034)	11.3 (7.20, 15.4)
	HDL	39 (1114)	-4.01 (-5.3, -2.73)	38 (1060)	-3.52 (-4.80, -2.25)
	LDL	32 (996)	7.93 (5.69, 10.2)	32 (996)	7.93 (5.69, 10.2)
	HOMA	5 (165)	0.12 (-0.05, 0.28)	5 (165)	0.12 (-0.05, 0.28)
Norgestimate	BMI	3 (41)	0.19 (-0.64, 1.01)	3 (41)	0.19 (-0.64, 1.01)
	Glucose	3 (41)	0.59 (-1.2, 2.39)	3 (41)	0.59 (-1.2, 2.39)
	TG	5 (100)	28.5 (25.5, 31.5)	5 (100)	28.5 (25.5, 31.5)
	HDL	3 (58)	2.58 (-1.78, 6.93)	3 (58)	2.58 (-1.78, 6.93)
	LDL	5 (83)	11.5 (3.82, 19.2)	5 (83)	11.5 (3.82, 19.2)
	HOMA	-	-	-	-
