# Supplementary material for: Effects of oral contraceptives on metabolic parameters in adult premenopausal women: a meta-analysis
Source: Endocr Connect. 2020 Sep 10;9(10):978–98. doi: 10.1530/EC-20-0423 (PMC7576645; doi:10.1530/EC-20-0423)
Supplement: Supplemental Figure 1. Funnel plots of mean weighted difference versus standard error of the weighted mean difference for metabolic outcomes in studies of oral contraceptives containing cyproterone. Egger´s and Begg´s tests were only calculated for outcomes in which there were at least 5 observation [file supplementary_figure_1.pdf]

Supplemental Figure 1. Funnel plots of mean weighted difference *versus* standard error of the weighted mean difference for metabolic outcomes in studies of oral contraceptives containing cyproterone. Egger´s and Begg´s tests were only calculated for outcomes in which there were at least 5 observations (studies).

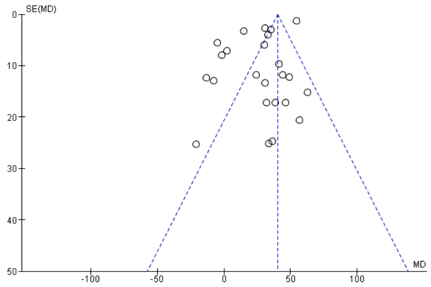

TG
Begg´s -0.07, p=0.36; Egger´s -0.10, p=0.62

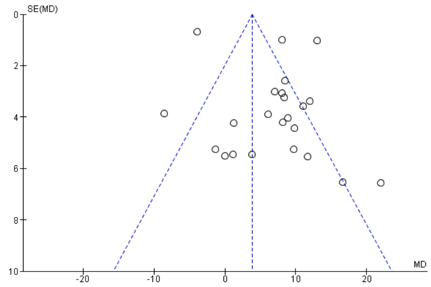

HDLc
Begg´s 0.17, p=0.45; Egger´s 0.20, p=0.35

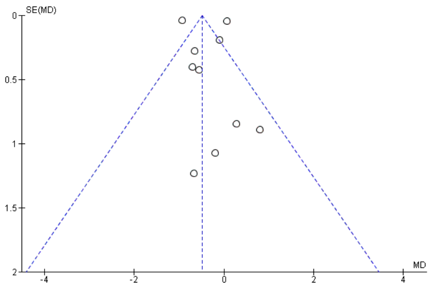

HOMA-IR
Begg´s 0.26, p=0.47; Egger´s 0.01, p=0.95

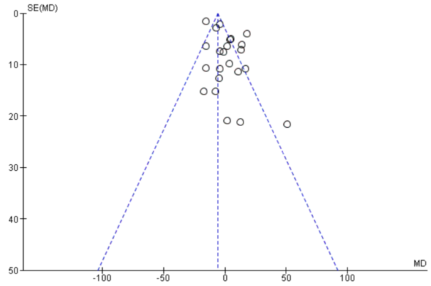

LDLc
Begg´s -0.06, p=0.77; Egger´s 0.24, p=0.25

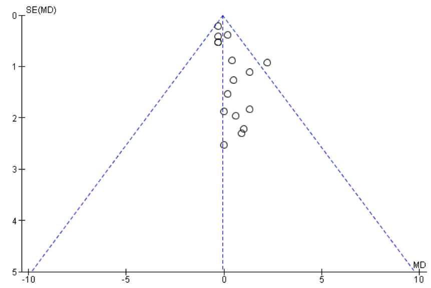

BMI
Begg´s 0.49, p=0.023; Egger´s 0.23, p=0.38

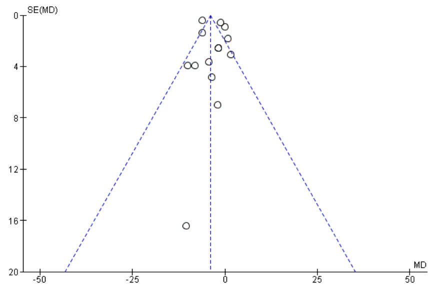

Glucose
Begg´s -0.38, p=0.09; Egger´s -0.50, p=0.03
