# Supplementary material for: Effects of oral contraceptives on metabolic parameters in adult premenopausal women: a meta-analysis
Source: Endocr Connect. 2020 Sep 10;9(10):978–98. doi: 10.1530/EC-20-0423 (PMC7576645; doi:10.1530/EC-20-0423)
Supplement: Supplemental Figure 2. Funnel plots of mean weighted difference versus standard error of the weighted mean difference for metabolic outcomes in studies of oral contraceptives containing desogestrel. Egger´s and Begg´s tests were only calculated for outcomes in which there were at least 5 observation [file supplementary_figure_2.pdf]

Supplemental Figure 2. Funnel plots of mean weighted difference *versus* standard error of the weighted mean difference for metabolic outcomes in studies of oral contraceptives containing desogestrel. Egger´s and Begg´s tests were only calculated for outcomes in which there were at least 5 observations (studies).

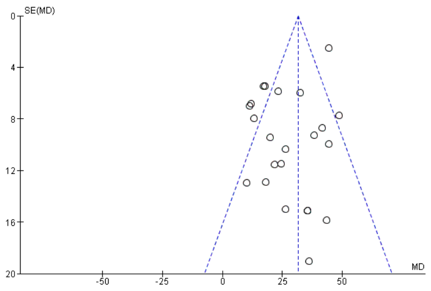

TG

Begg´s 0.17, p=0.44; Egger´s 0.15, p=0.48

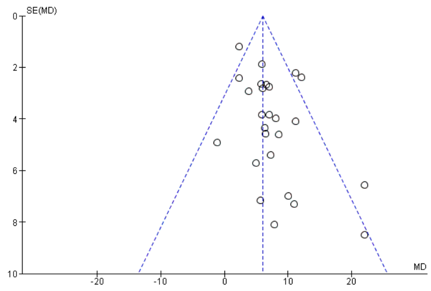

HDLc

Begg´s -0.14, p=0.26; Egger´s 0.20, p=0.88

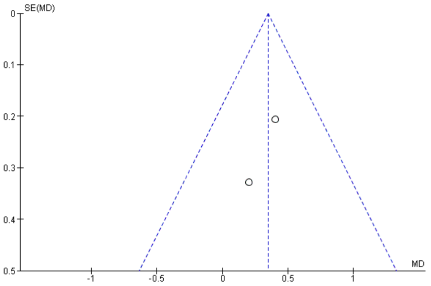

HOMA-IR

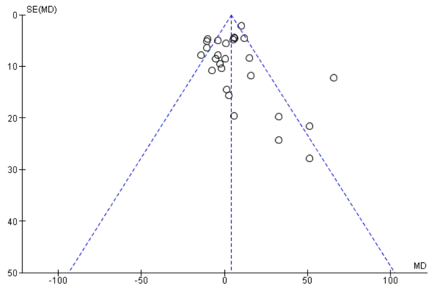

LDLc

Begg´s 0.38, p=0.050; Egger´s 0.64, p<0,001

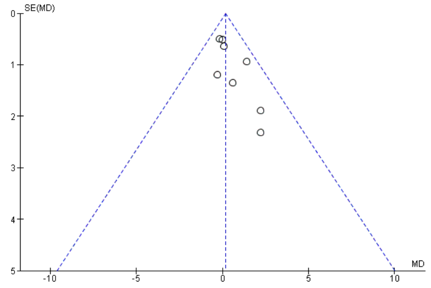

BMI

Begg´s 0.68, p=0.062; Egger´s 0.66, p=0.07

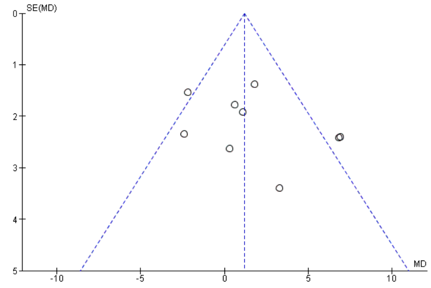

Glucose

Begg´s -0.03, p=0.47; Egger´s -0.54, p=0.30
