# Supplementary material for: Effects of oral contraceptives on metabolic parameters in adult premenopausal women: a meta-analysis
Source: Endocr Connect. 2020 Sep 10;9(10):978–98. doi: 10.1530/EC-20-0423 (PMC7576645; doi:10.1530/EC-20-0423)
Supplement: Supplemental Figure 3. Funnel plots of mean weighted difference versus standard error of the weighted mean difference for metabolic outcomes in studies of oral contraceptives containing drospirenone. Egger´s and Begg´s tests were only calculated for outcomes in which there were at least 5 observatio [file supplementary_figure_3.pdf]

Supplemental Figure 3. Funnel plots of mean weighted difference *versus* standard error of the weighted mean difference for metabolic outcomes in studies of oral contraceptives containing drospirenone. Egger´s and Begg´s tests were only calculated for outcomes in which there were at least 5 observations (studies).

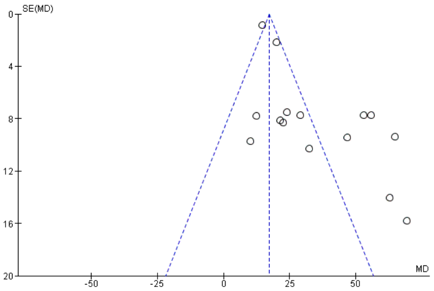

TG

Begg´s 0.45, p=0.10; Egger´s 0.62, p=0.01

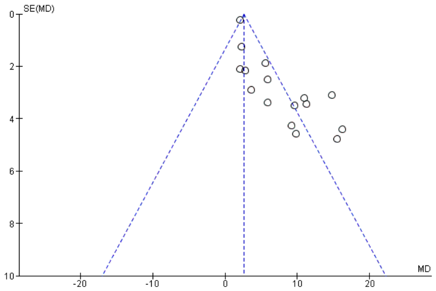

HDLc

Begg´s 0.85, p<0.001; Egger´s 0.88, p<0.001

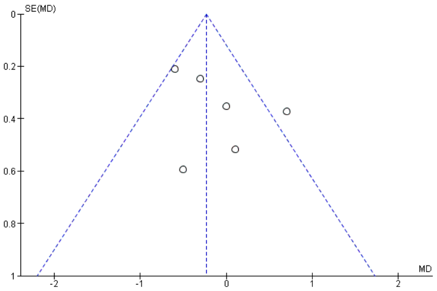

HOMA-IR

Begg´s 0.37, p=0.18; Egger´s 0.12, 0.66

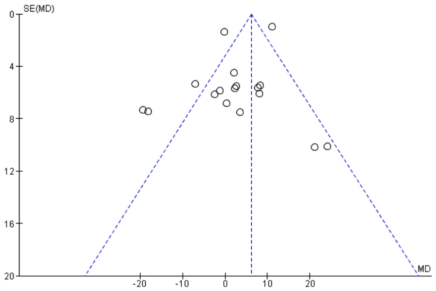

LDLc

Begg´s 0.05, p=0.85; Egger´s 0.14, p=0.63

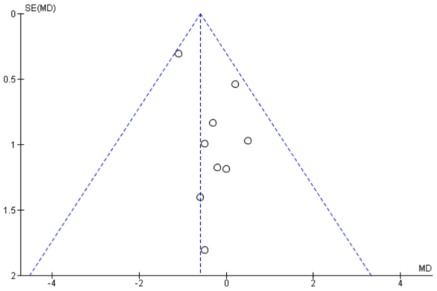

BMI

Begg´s -0.15, p=0.35; Egger´s -0.15, p=0.34

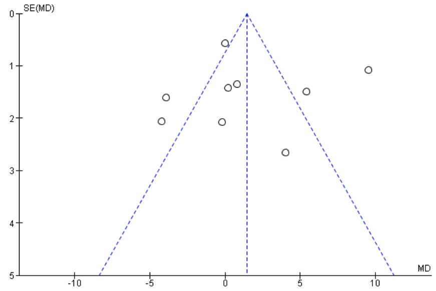

Glucose

Begg´s -0.32, p=0.20; Egger´s -0.10, p=0.39
