# Supplementary material for: Effects of oral contraceptives on metabolic parameters in adult premenopausal women: a meta-analysis
Source: Endocr Connect. 2020 Sep 10;9(10):978–98. doi: 10.1530/EC-20-0423 (PMC7576645; doi:10.1530/EC-20-0423)
Supplement: Supplemental Figure 4. Funnel plots of mean weighted difference versus standard error of the weighted mean difference for metabolic outcomes in studies of oral contraceptives containing gestodene. Egger´s and Begg´s tests were only calculated for outcomes in which there were at least 5 observations  [file supplementary_figure_4.pdf]

Supplemental Figure 4. Funnel plots of mean weighted difference *versus* standard error of the weighted mean difference for metabolic outcomes in studies of oral contraceptives containing gestodene. Egger´s and Begg´s tests were only calculated for outcomes in which there were at least 5 observations (studies).

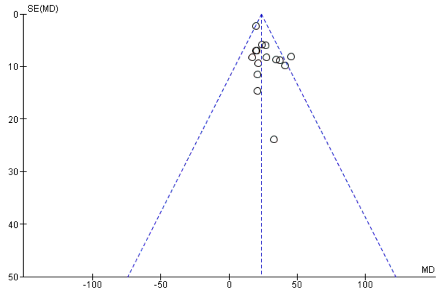

TG

Begg´s -0.01, p=0.50; Egger´s -0.15, p=0.30

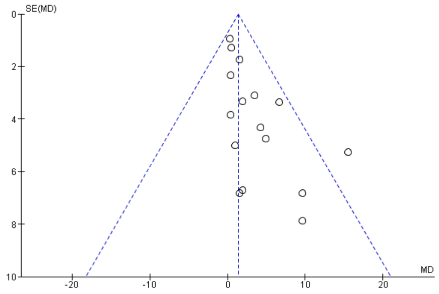

HDLc

Begg´s 0.52, p=0.04; Egger´s 0.42, p=0.10

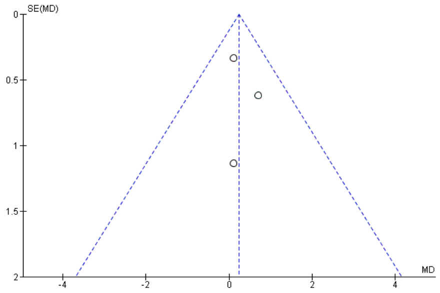

BMI

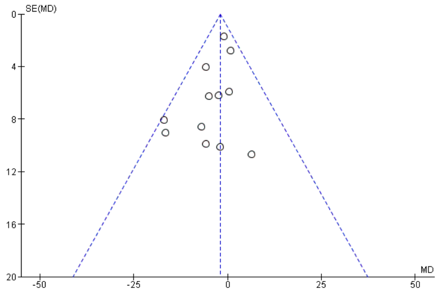

LDLc

Begg´s -0.20, p=0.27; Egger´s -0.26, p=0.21

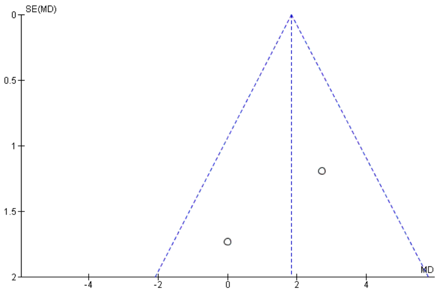

Glucose
