# Supplementary material for: Effects of oral contraceptives on metabolic parameters in adult premenopausal women: a meta-analysis
Source: Endocr Connect. 2020 Sep 10;9(10):978–98. doi: 10.1530/EC-20-0423 (PMC7576645; doi:10.1530/EC-20-0423)
Supplement: Supplemental Figure 5. Funnel plots of mean weighted difference versus standard error of the weighted mean difference for metabolic outcomes in studies of oral contraceptives containing levonorgestrel. Egger´s and Begg´s tests were only calculated for outcomes in which there were at least 5 observat [file supplementary_figure_5.pdf]

Supplemental Figure 5. Funnel plots of mean weighted difference *versus* standard error of the weighted mean difference for metabolic outcomes in studies of oral contraceptives containing levonorgestrel. Egger´s and Begg´s tests were only calculated for outcomes in which there were at least 5 observations (studies).

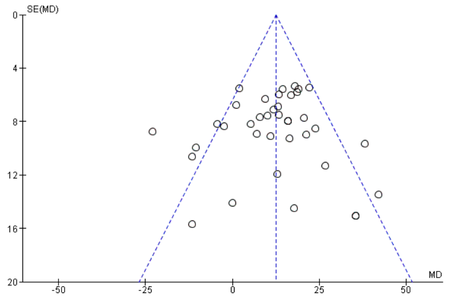

TG

Begg´s 0.00, p=0.99; Egger´s 0.10, p=0.55

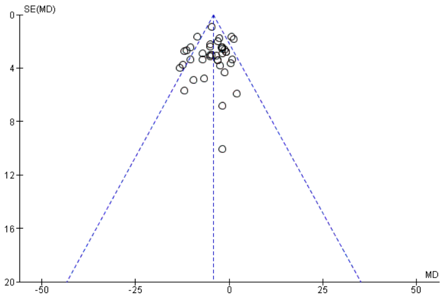

HDLc

Begg´s -0.15, p=0.18; Egger´s -0.14, p=0.19

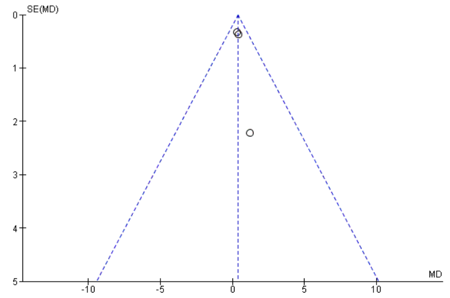

HOMA

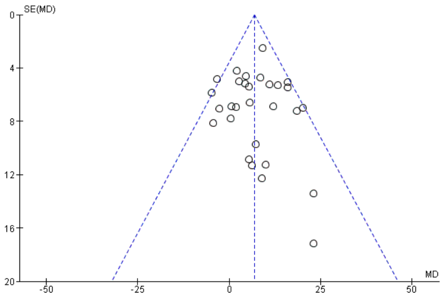

LDLc

Begg´s 0.44, p=0.007; Egger´s 0.48, p=0.002

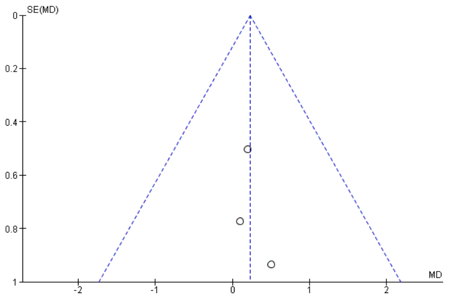

BMI

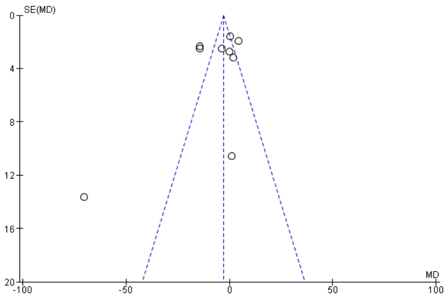

Glucose
