# Supplementary material for: Effects of oral contraceptives on metabolic parameters in adult premenopausal women: a meta-analysis
Source: Endocr Connect. 2020 Sep 10;9(10):978–98. doi: 10.1530/EC-20-0423 (PMC7576645; doi:10.1530/EC-20-0423)

Supplemental Figure 6. Trim-and-fill analysis of the four intervention-outcome pairs that showed indication of publication bias. Upper-left: Desogestrel and LDLc, upper-right: Drospirenone and TG, lower-left: Drospirenone and HDLc, lower-right: Levonorgestrel and LDLc. Only the modification of HDLc after drospirenone use was affected by publication bias adjustment.

Desogestrel and LDLc

Studies	Effect Size	[95% Conf.	Interval]
Observed	4.343	-1.218	9.904
Observed + Imputed	4.343	-1.218	9.904

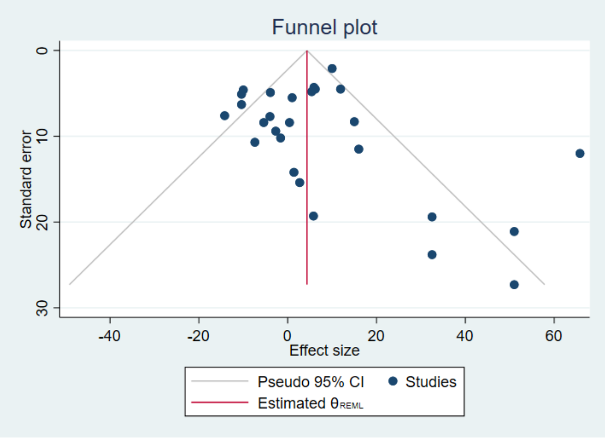

Drospirenone and TG

Studies	Effect Size	[95% Conf.	Interval]
Observed	34.204	24.275	44.132
Observed + Imputed	30.242	19.665	40.818

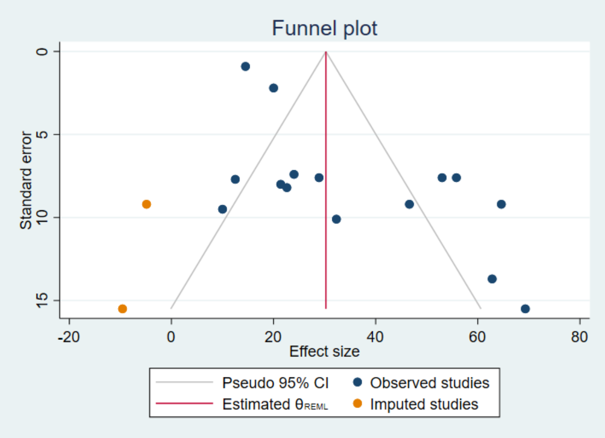

Drospirenone and HDLc

Studies	Effect Size	[95% Conf.	Interval]
Observed	7.759	5.136	10.382
Observed + Imputed	3.559	-0.095	7.213

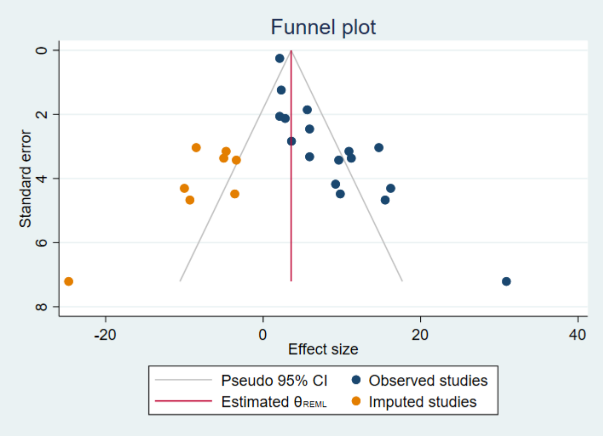

Levonorgestrel and LDLc

Studies	Effect Size	[95% Conf.	Interval]
Observed	7.801	5.454	10.148
Observed + Imputed	7.801	5.454	10.148

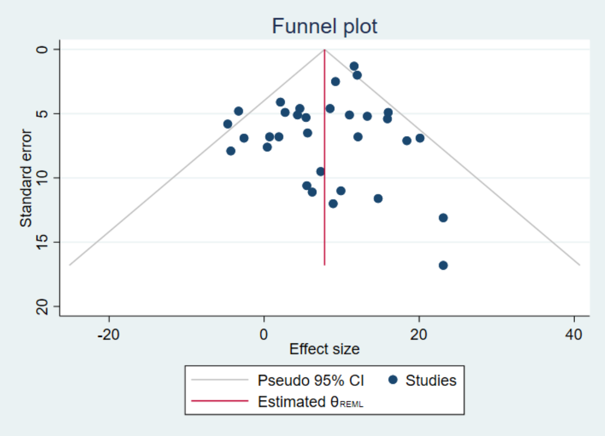

Supplement: Supplemental Figure 6. Trim-and-fill analysis of the four intervention-outcome pairs that showed indication of publication bias. Upper-left: Desogestrel and LDLc, upper-right: Drospirenone and TG, lower-left: Drospirenone and HDLc, lower-right: Levonorgestrel and LDLc. Only the modification of HDLc  [file supplementary_figure_6.pdf]
